# Flavonoids in Lotus Stamen Extract Inhibit High Glucose-Induced Intracellular Glycation in Fibroblasts by Upregulating the Expression of Glyoxalase 1 and Alleviating Oxidative Stress

**DOI:** 10.3390/antiox14040392

**Published:** 2025-03-26

**Authors:** Wenge Zheng, Ruiling Chen, Kewei Xu, Rui Wang, Zhiyuan Wang, Huijuan Li, Yuyo Go, Xihui Chan, Qing Huang, Jianxin Wu

**Affiliations:** 1 Skin Health and Cosmetic Development & Evaluation Laboratory, China Pharmaceutical University, Nanjing 210009, China3124024175@stu.cpu.edu.cn (R.W.); 3322020794@stu.cpu.edu.cn (Z.W.);; 2 Department of Engineering Science, University of Oxford, Oxford OX1 3PJ, UK; 3 Department of Medicine, Waikato Hospital, Hamilton 3204, New Zealand

**Keywords:** lotus stamens, flavonoids, skin glycation, dicarbonyl agents, glyoxalase 1

## Abstract

Glycation is a process in which reducing sugars bind to proteins, resulting in the formation of advanced glycation end products (AGEs). These AGEs accumulate in the skin, promote excessive collagen crosslinking, and disrupt the extracellular matrix (ECM), impairing normal cellular functions and contributing to skin aging. To evaluate the anti-glycation efficacy of lotus stamen extract (LSE), we employed the BSA–fructose system and a high glucose (HG)-induced fibroblast glycation model. The results demonstrated that LSE effectively inhibited cellular glycation and also exhibited anti-inflammatory, antioxidative, and anti-senescent effects in HG-induced human skin fibroblasts (HSF). Further investigation into the anti-glycation mechanism and component analysis of the lotus stamen ethyl acetate extract (LSEE) led to the identification of 15 flavonoids. The anti-glycation results indicated that these flavonoids are likely the primary active constituents in LSE. Mechanistic studies revealed that GLO1 plays a crucial role in cellular resistance to glycation, and LSEE enhanced GLO1 expression through the Nrf2/Keap1 pro-survival pathway, thereby mitigating intracellular AGE production. In summary, LSEE and its multiple flavonoid components exhibit potent intracellular anti-glycation activity and present significant potential to be developed as a natural and organic product for cosmetic and healthcare applications.

## 1. Introduction

Aging is an inevitable physiological process, with glycation recognized as one of the most important contributors to skin aging [1]. Glycation is often defined as a non-enzymatic chemical reaction between reducing sugars and proteins, leading to skin manifestations, such as wrinkles, reduced elasticity, dullness, and yellowing [2]. Glycation can occur both extracellularly and intracellularly in the skin, resulting in the formation of advanced glycation end products (AGEs), which disrupt normal cellular physiology through their reaction process and subsequent products. AGEs are primarily formed through the Maillard reaction, where reducing sugars react with free amino groups (including proteins, nucleic acids, and lipids) to form the unstable aldehyde–amide intermediates (known as Schiff’s bases), which are subsequently rearranged to form Amadori products and degraded into various reactive dicarbonyl compounds (glyoxal (GO), methylglyoxal (MGO), and 3-deoxyglucosone (3-DG)) [3,4]. These highly reactive dicarbonyls re-react with free amino groups in proteins to form fluorescent, irreversible, yellow-brown compounds. Glycation may start early in life, with glycated collagen accumulating at a rate of 3.7% per year, a process that accelerates in all tissues when sugars in the skin are elevated and further stimulated by ultraviolet [5].

Skin glycation occurs through the interaction between glucose and amino acids found in collagen and elastin, the primary structural proteins that support the dermal extracellular matrix (ECM). This interaction leads to the formation of AGEs and the development of crosslinks, which can compromise skin elasticity and contribute to aging [6]. ECM is a complex network of non-cellular structures, including hyaluronic acid, fibronectin, collagen and elastin, which are mainly produced by fibroblasts and provide mechanical strength and elastic resilience to the skin [7]. Collagen is the most abundant protein in the ECM and is particularly susceptible to glycation and the accumulation of AGEs due to its slow turnover rate [8]. Multiple AGE intermediates, including glucosepane, fructosyl-lysine, Nε-(carboxymethyl) lysine (CML), pentosidine, and Nε-(carboxyethyl) lysine (CEL), have been identified at elevated levels in the skin [9]. CML, one of the major AGE intermediates, accumulates in dermal collagen, elastin, and glycosaminoglycans [10], and is also the main target of waveform protein in fibroblasts [11]. These AGEs accumulate in the skin with age, reduce the natural defense against free radicals in the body, and promote the formation of crosslinks which are hard to repair [12].

Normally, cells also experience glycation due to metabolic processes, such as glycolysis and lipid oxidation, which continuously generate reducing sugars and highly reactive dicarbonyls (GO, MGO and 3-DG) [13]. These dicarbonyl compounds are potent glycating agents for proteins and nucleotides [14]. Glycation caused by MGO and GO may represent up to 65% of glycation events, which inflict adverse effects on cellular physiological processes [15]. MGO can form covalent adducts with cysteine, arginines, and lysines, primarily forming MGO-derived hydroimidazolone (MG-H1), CEL, and methylglyoxal-lysine dimer (MOLD). GO forms similar glycosylation adducts, predominantly GO-derived hydroimidazolone (G-H1), CML and glyoxal-lysine dimer (GOLD) [16]. DNA is also sensitive to glycosylation modifications, with GO inducing DNA strand breaks and MGO producing extensive DNA–protein crosslinks [17].

Fibroblasts, as the primary repair and collagen-secreting cells in the dermis, are essential for maintaining the normal structure and physiological functions of skin, and they also play an important role in the degradation and accumulation of AGEs in the skin [18]. AGEs can induce fibroblast senescence and apoptosis. The binding of AGEs to receptors (RAGE) on cell membranes activates NF-κB and mitogen-activated protein kinase (MAPK) signaling pathways [19], which leads to the production of intracellular reactive oxygen species (ROS), inflammatory cytokines (interleukin 6, IL-6 and tumor-necrosis factor alpha, TNF-α), and metalloproteinase (matrix metalloproteinase 1, MMP1; matrix metalloproteinase 2, MMP2; and matrix metalloproteinase 9, MMP9) production [20]. ROS generation further promotes the production of reactive aldehydes and their derivatives, namely advanced lipid peroxidation (ALE) and advanced glycation (AGE) end products, by inducing lipid peroxidation and glycoxidation [21].

Glucose, the primary energy source for mammalian cells, fuels glycolysis and the tricarboxylic acid (TCA) cycle [22], and its AGE formation rate is much slower than other natural sugars (e.g., glyceraldehyde-3-PO 4, glucose-6-PO 4, and fructose) [23]. The persistence of high glucose (HG) leads to glucose autoxidation, protein glycation, and the activation of metabolic pathways, including glycolysis, the hexosamine pathway, and polyol metabolism. These processes contribute to intracellular inflammation and oxidative stress [24,25] and promote premature cellular senescence [26].

*Nelumbo nucifera* Gaertn., commonly known as lotus, has a long history of edible and medicinal use in China, and its various parts, such as seeds, stamens, and leaves, can be used as food, nutrients, or to treat a variety of ailments, including in anti-diabetic, antioxidant, anti-inflammatory, and anti-tumor medicine [27,28]. Lotus contains numerous beneficial compounds, such as flavonoids, alkaloids, polyphenols, polysaccharides, and amino acids [29]. Research has demonstrated that the flavonoids in lotus exhibit hypoglycemic [30] and α-amylase inhibitory activities, suggesting their potential as preventive agents against type 2 diabetes [31,32]. Compared to other parts of lotus, the stamens are particularly rich in flavonoids [33,34], including kaempferol, syringetin, quercetin, isorhamnetin, etc., as well as their glycosides, all of which display significant antioxidant properties [35]. However, there is limited research related to the anti-glycation effect of lotus stamens.

To investigate cellular glycation-related events, we established an HG-induced human skin fibroblast (HSF) glycation model and demonstrated that lotus stamen extract (LSE) and its flavonoids could mitigate HG-induced damage in HSFs by measuring intracellular markers, such as CML, AGEs fluorescence, RAGE, and NF-κB, as well as markers of cellular senescence and oxidative stress. Furthermore, glyoxalase 1 (GLO1) is a key enzyme involved in the detoxification of dicarbonyl scavenging. GLO1 was found to be upregulated by lotus stamen ethyl acetate extract (LSEE) and its major flavonoids (hyperoside (quercetin-3-O-galactoside) and astragalin (kaempferol-3-O-glucoside)) and may be mediated through the activation of the Nrf2/Keap1 pathway. These findings provide insights into the anti-glycation effect of LSE and its underlying mechanisms, which are important for advancing cellular anti-glycation research and in the discovery of novel natural anti-glycation products.

## 2. Materials and Methods

### 2.1. Materials and Chemicals

Fetal bovine serum (FBS), DMEM, penicillin-streptomycin solution, and trypsin-EDTA were obtained from Thermo Fisher Scientific (Waltham, MA, USA). Aldehydo-D-glucose was obtained from the China National Pharmaceutical Group Corporation (Beijing, China); an Interleukin-6 (IL-6, human) ELISA kit was obtained from MultiSciences (Lianke) Biotech Co., Ltd. (Hangzhou, China). A CML (human) ELISA kit, AGEs (human) ELISA kit, and GLO1 (human) ELISA kit were obtained from Jiangsu Meimian Industrial Co., Ltd. (Yancheng, China). 3-(4,5-dimethylthiazol-2-yl)-2,5-diphenyltetrazolium bromide (MTT) and RIPA buffer were obtained from Solarbio Science & Technology Co., Ltd. (Beijing, China). A lactate dehydrogenase (LDH) assay kit, glutathione (GSH) assay kit, superoxide dismutase (SOD, WST-1 method) assay kit, and malondialdehyde (MDA) assay kit were obtained from Nanjing Jiancheng Bioengineering Institute (Nanjing, China). L-Ascorbic acid (VC) was obtained from Xilong Scientific Co., Ltd. (Shenzhen, China). RNA-easy, 4× gDNA wiper Mix, 5× HiScript III qRT SuperMix, and 2× ChamQ SYBR qPCR Master Mix (Low ROX Premixed) were obtained from Vazyme Biotech Co., Ltd. (Nanjing, China). Aminoguanidine hydrochloride (AG, purity ≥ 98%) was obtained from Aladdin Biochemical Technology Co., Ltd. (Shanghai, China). Metformin hydrochloride (MF) was obtained from Sangon Biotech Co., Ltd. (Shanghai, China). Quercetin (purity ≥ 98%), kaempferol (purity ≥ 97%), kaempferol 3-O-glucoside (purity ≥ 98%), quercetin 3-D-galactoside (purity ≥ 97%), methylglyoxal (MGO, 32% solution), S-p-Bromobenzylglutathione cyclopentyldiester (SpBrGSHCp2, Purity ≥ 98%), and 2,2-Diphenyl-1-picrylhydrazyl (DPPH) were obtained from Mackin Biochemical Co., Ltd. (Shanghai, China). A reactive oxygen species (ROS) assay kit and BCA protein assay kit were obtained from Beyotime Biotechnology (Shanghai, China). Sodium phosphate buffer solution (PBS, PH 7.0 and PH 7.2) was obtained from Leagene Biochemical Technology Co., Ltd. (Beijing, China); the anti-p-NF-κb, anti-NF-κb, anti-p38 MAPK, anti-p-p38 MAPK, p53, and p16-INK4A were obtained from Cell Signaling Technology (Danvers, MA, USA). The anti-RAGE and anti-p21 were obtained from Abcam (Cambridge, UK). Anti-β-actin was obtained from Zen-Bioscience Co., Ltd. (Chengdu, China). Goat anti-rabbit IgG HRP was obtained from Biosharp Life Sciences Co., Ltd., (Beijing, China).

### 2.2. Human Skin Fibroblasts (HSFs)—Culture and Treatment

Human skin fibroblasts (HSFs) were isolated from healthy foreskin tissues provided by Sir Run Run Hospital of Nanjing Medical University. The study protocol was approved by the Research Ethics Committee of Sir Run Run Hospital, Nanjing Medical University (ethical approval number: 2024-SR-056), and informed consent was obtained from all foreskin tissue donors. HSFs were maintained in Dulbecco’s modified Eagle’s medium (DMEM) containing 25 mM of glucose, supplemented with 10% fetal bovine serum (FBS) and 1% penicillin-streptomycin. Cells were incubated at 37 °C in a humidified atmosphere of 5% CO_2_. HSFs at passages 4–6 were seeded into 96-well (5 × 10^3^ cells/well) or 6-well (1.5 × 10^5^ cells/well) plates and cultured for 24 h, then treated with 25, 35, 75, or 125 mM of glucose for 48 h for the study of the effects of glucose on the HSFs. Here, 25 mM of glucose was chosen as the normal condition, since fibroblasts have already adapted their metabolic characteristics to the standard high-glucose culture system (25 mM DMEM) used in our laboratory. For the study of the protective effects of LSE or other drugs on HG-induced HSF glycation, HSFs at passages 4–6 were seeded into 96-well or 6-well plates and cultured for 24 h. The HSFs were pretreated with LSE (0.1, 1, or 10 μg/mL) or other drugs for 2 h; then, the culture medium was supplemented with glucose to a final concentration of 125 mM (HG model condition) and incubated for 48 h.

### 2.3. AGE Solution Preparation

A total of 600 mg of BSA powder (20 mg/mL) and 54.1 mg of glyceraldehyde powder (20 mM) were placed into a 50 mL centrifuge tube. Then, 30 mL of sterile PBS was added, and the mixture was thoroughly dissolved by shaking. The solution was filtered through a 0.2 μm bacterial filtration membrane, then placed in an incubator at 37 °C, protected from light, and allowed to react for one week. One week later, the AGE solution was dialyzed three times with PBS using a semi-permeable membrane (pore size is 3500 Da), for 6 h each time. In a clean bench, the AGE solution was filtered with a 0.2 μm bacterial filtration membrane and then stored at −20 °C. The synthetic AGE solution was characterized using fluorescence and ultraviolet methods.

### 2.4. Cell Viability Assay

The HSFs were seeded into 96-well plates and treated as described in Section 2.2. After treatment, the cell supernatant was removed and 10 μL of MTT solution (5 mg/mL) was added to each well. The plate was placed at 37 °C for 4 h to allow for formazan formation. Then, the DMSO was used to dissolve formazan, and the plate was measured at 570 nm by using the SpectraMax 190 microplate reader (Molecular Devices, San Jose, CA, USA).

### 2.5. Glucose Concentration Assay

The HSFs were seeded into 96-well plates and treated as described in Section 2.2. The cell supernatant was collected for glucose concentration detection. According to the protocol of the glucose assay kit (GOD-POD, colorimetric method) (Yuanye Bio-Technology Co., Ltd., Shanghai, China), 10 μL of cell supernatant was incubated with 300 μL of GOD-POD working fluid at 37 °C for 15 min. After incubation, the absorbance was measured at 505 nm using a SpectraMax 190 microplate reader (Molecular Devices, San Jose, CA, USA).

(1) $$\mathrm{Glu}\;(\mathrm{mmol}/L)=A_{assay}/A_{standard}\;\times \;{Glu}_{standard}(5\;mmol/L)$$

(2) $$\mathrm{Glucose}\;\mathrm{consumption}\;(\mathrm{mmol}/L)={Glu}_{48h}-{Glu}_{0h}\;or\;{Glu}_{96h}-{Glu}_{48h}$$

### 2.6. β-Galactosidase Assay

Senescent cells were identified using a senescence-associated β-galactosidase kit (Solarbio Science & Technology Co., Ltd., Beijing, China). The HSFs were seeded into 96-well plates and treated as described in Section 2.2. After washing with PBS three times, the HSFs were fixed with fixative solution at room temperature for 15 min and washed with PBS three times. The cells were then incubated with freshly prepared staining buffer overnight at 37 °C. The following day, photographs were taken using a fluorescence inversion microscope (Mshot, Guangzhou, China).

### 2.7. LDH, GSH, SOD, and MDA Assays

The HSFs were seeded into 6-well plates and treated as described in Section 2.2. The HSFs were collected, and the levels of LDH, GSH, and MDA, as well as SOD activity, were measured using the corresponding kits according to the manufacturer’s protocols.

### 2.8. Assay of ROS

The HSFs were seeded into 6-well plates and treated as described in Section 2.2. The HSFs were washed three times with PBS, and DCFH-DA was diluted with serum-free medium (1:1000). Then, 1 mL of the diluted DCFH-DA was added to each well and incubated in a cell culture incubator at 37 °C for 20 min. The cells were excited by UV light under a fluorescence microscope, and the green fluorescence was observed and photographed. The fluorescence intensity was detected by a CytoFLEX flow cytometer (Beckman Coulter, Brea, CA, USA).

### 2.9. ELISA Assay of AGEs, CML, IL-6, and GLO1

The HSFs were seeded into 6-well plates and treated as described in Section 2.2. The cells were washed three times with PBS and lysed with RIPA buffer (supplemented with 1% PMSF) on ice. After centrifuging at 2–8 °C for 20 min (2000–3000 r/min), the supernatant was collected for the detection of AGEs, CML, and GLO1 using ELISA kits, following the manufacturer’s protocols. The cell supernatant (culture medium) was centrifuged at 2–8 °C for 20 min (2000–3000 r/min) for the detection of IL-6. Protein concentration was quantified using the BCA assay. All samples were adjusted to ensure they had the same protein concentration. Each experiment was repeated at least three times.

### 2.10. Fluorescence Detection

The HSFs were seeded into 96-well or 6-well plates and treated as described in Section 2.2. The cell supernatant was collected and added to a 96-well plate (300 μL per well) for detection. The supernatant fluorescence was measured using a Varioskan flash spectral scanning multimode reader (Thermo Fisher Scientific, Waltham, MA, USA) with excitation at 370 nm and emission at 440 nm. The cells were collected, and cellular fluorescence was detected using a CytoFLEX flow cytometer (Beckman Coulter, CA, USA).

### 2.11. Measurement of p16, p21, RAGE, NF-κB, GLO1, Nrf2, and Keap1 mRNA Expression

The HSFs were seeded into 6-well plates and treated as described in Section 2.2. The HSF cells were washed with PBS and lysed using RNA-easy. The RNA pellets were obtained after purification with isopropanol and 75% ethanol, and the RNA concentration was determined using an ultra-micro spectrophotometer (Nano-100). Subsequently, DNA was removed from the samples, and reverse transcription was performed to synthesize cDNA. A total of 1 µg of cDNA is obtained for each sample. Real-time PCR was performed using the BIOER LineGene 9600 Plus PCR device (Bioer Technology, Hangzhou, China). The mRNA level was normalized to the β-actin expression in each sample. The sequences of all primers are shown in Table 1.

### 2.12. Western Blot Analysis

The HSFs were seeded into 6-well plates and treated as described in Section 2.2. The HSFs were collected and lysed with RIPA buffer (supplemented with 1% PMSF and 10% phosphatase inhibitor) on ice. Protein concentration was quantified using the BCA assay. The cell lysates were separated by SDS-PAGE, and the proteins were transferred onto a polyvinylidene fluoride (PVDF) membrane. The membranes were then exposed to the following appropriate antibodies overnight: β-actin (1:2500), p16-INK4A (1:1000), p21 (1:1000), p53 (1:1000), RAGE (1:1000), NF-κb (1:1000), p-NF-κb (1:1000), p38 MAPK (1:1000), and p-p38 MAPK (1:1000). The next day, the membranes were incubated with goat anti-rabbit IgG HRP (2:5000) and visualized by chemiluminescence. Images were captured using an automatic chemiluminescence imaging analysis system (Tanon 5200, Shanghai, China), and grayscale analysis of the protein bands was performed using ImageJ version 1.54j software. Each experiment was repeated at least three times.

### 2.13. Lotus Stamen Sample Preparation

The lotus stamen herbs (*N. nucifera* Gaertn., purchased from the Bozhou herbal medicine market) were crushed using a wall breaker and sieved through a 30-mesh sieve. The crushed lotus herbs were immersed with 30% ethanol aqueous solution (material-to-liquid ratio of 1:20) and subjected to ultrasonic extraction (100 W) at room temperature for 1 h. This process was repeated twice, and the filtrates were combined and centrifuged at 3500 rpm and 4 °C for 30 min. The supernatants were then filtered and concentrated at 60 °C and 80 rpm using a rotary evaporator. Finally, the extraction solution was lyophilized using a freeze dryer and stored at −80 °C.

### 2.14. Sample Preparation of Different Polar Parts

The concentrated crude extract of lotus stamens was fully dissolved in distilled water and transferred to a separatory funnel. It was then sequentially extracted with double the volume of petroleum ether, ethyl acetate, and n-butanol, following the order of polarity from low to high. Each solvent was applied for three rounds of extraction. This procedure yielded four fractions: petroleum ether extract, ethyl acetate extract, n-butanol extract, and water extract. After concentrating each fraction using a rotary evaporator, the extracts were freeze-dried and were then stored at −80 °C.

### 2.15. Total Flavonoid Content (TFC) Assay

The dried powders of lotus stamens and the different polar parts of the stamens were diluted to 1 mg/mL with 60% ethanol. Then, 100 μL of each sample was transferred into a centrifuge tube, and 400 μL of 60% ethanol and 30 μL of 5% sodium nitrite solution were added. After 6 min, 30 μL of 10% aluminum nitrate aqueous solution was added. After another 6 min, 400 μL of 4% sodium hydroxide aqueous solution was added, and the mixture was allowed to stand for 15 min. The absorbance was measured at 510 nm, and the total flavonoids value was calculated according to the linear regression equation.

### 2.16. Total Phenol Content (TPC) Assay

The dried powders of lotus stamens and the different polar parts of the stamens were diluted to 1 mg/mL with 60% ethanol. Then, 10 μL of each sample was transferred into a centrifuge tube. To this, 590 μL of purified water, 100 μL of Folin-Ciocalteu reagent (Folinol solution), and 300 μL of 7.5% Na_2_CO_3_ solution were added. After incubating in a water bath at 45 °C for 1 h and 50 min, the absorbance was measured at 765 nm. The total polyphenol content was calculated according to the standard curve.

### 2.17. DPPH Free Radical Scavenging Activity

Different concentrations of the sample solution (100 μL each) were prepared and mixed with 100 μL of the 100 μg/mL DPPH solution. The mixture was incubated in the dark for 30 min. Here, 95% ethanol was used as the blank control group, and ascorbic acid (VC) was used as the positive control. The absorbance was measured at 517 nm and each set of experiments was paralleled three times. The DPPH radical clearance rate was calculated as follows:

(3) $$\mathrm{DPPH}\;\mathrm{clearance}\;\mathrm{rate}=\left(1-\frac{A_{1}-A_{2}}{A_{0}}\right)\;\times \;100\%$$

where A_1_ is 100 µL of sample solution + 100 µL of DPPH (experimental group), A_2_ is 100 µL of sample solution + 100 µL of DPPH (control group), and A_0_ is 100 µL sample solution + 100 µL 95% ethanol.

### 2.18. BSA–Fructose System Anti-Glycation Assay

The dried powders of stamens and the different polar parts of the stamens were diluted to 1 mg/mL with PBS. BSA (30 mg/mL, diluted with PBS) solution, fructose (1.5 mol/L, diluted with PBS) solution, and thimerosal sodium (0.01%, diluted with PBS) were prepared. Then, 70 μL of the sample solution was mixed with 70 μL of fructose solution and incubated at 37 °C for 2 h. Then, 70 μL of BSA solution was added to the mixture, and the mixed sample solution was incubated in an incubator at 37 °C for 7 days. Fluorescence was measured using a fluorescence spectrophotometer (enzyme labeler), with an excitation wavelength of 370 nm and an emission wavelength of 440 nm. Each set of experiments was repeated three times. Equation (4) is as follows:

(4) $$\mathrm{Inhibition}\;\mathrm{rate}=(1-\frac{F_{A}-F_{0}}{F_{B}})\;\times \;100\%$$

where F_A_ is 70 µL of sample solution + 70 µL of fructose (1.5 mol/L), where 70 μL BSA was added after the reaction at 37 °C for 2 h. F_B_ is 70 µL thimerosal sodium (0.01%) + 70 µL fructose (1.5 mol/L), where 70 μL BSA was added after the reaction at 37 °C for 2 h. F_0_ is 70 µL of sample solution + 70 µL thimerosal sodium (0.01%), where 70 μL BSA was added after the reaction at 37 °C for 2 h.

### 2.19. Chromatography and Mass Spectrometry Conditions

#### 2.19.1. Chromatographic Conditions

An Acclaim120 c18 column (250 mm × 46 μm, 5 μm) was used. The column temperature was 37 °C, the injection volume was 20 μL, the flow rate was 1.0 mL/min, the UV detection wavelength was 266 nm, and the mobile phase was 1% acetic acid in water (A) –methanol (B). The elution conditions in the negative ion mode were as follows: 0–10 min, 25% B; 10–13 min, 25% B—35% B; 13–23 min, 35% B; 23–24 min, 35% B–40% B; 24–50 min, 40% B.

#### 2.19.2. Mass Spectrometry Conditions

The ESI ion source was used, with a primary mass spectrometry scanning range of 50–1700 *m*/*z*, a nebulizing gas (psi) of 35, an ion source voltage (V) of 5000, an ion source temperature of 500 °C, a de-clustering voltage (V) of 100; and a collision energy (ev) of 10. The secondary mass spectrometry scanning range was 50–1250 *m*/*z*, the de-clustering voltage (V) was 100, the collision energy (ev) was 40, and the collision voltage swing (ev) was 20.

### 2.20. GLO1 Activity Assay

The HSFs were seeded into 6-well plates and treated as described in Section 2.2. Subsequently, the cells were collected and suspended in 200 μL of 10 mM sodium phosphate buffer (pH 7.0) at 4 °C. The suspension was sonicated at 100 W for 30 s to lyse the cells. Cytosolic fibers and cell membranes were precipitated by centrifugation at 1.5 × 10^4^ *g* at 4 °C for 30 min. After centrifugation, the supernatant was carefully discarded, and the cell pellet was kept on ice for further analysis. To prepare the hemithio-acetal solution, 9.6 mL of 0.1 M sodium phosphate buffer (pH 7.2), 0.2 mL of 100 mM glutathione (GSH) solution, and 0.2 mL of 100 mM methylglyoxal (MG) solution were mixed, and the reaction mixture was incubated at 37 °C for 20 min in a magnetic stirrer [36,37]. For the Glo1 activity assay, 950 μL of the hemithio-acetal solution was mixed with 50 μL of the cell precipitate (the cell precipitate was resuspended and diluted in sodium phosphate buffer), and the mixture was incubated at 37 °C for 5 min. The absorbance was measured at 240 nm, for which the change in molar absorption coefficient Δε240 = 2.86 Mm^−1^·cm^−1^. Protein concentration was quantified using the BCA assay, and all samples were adjusted to ensure that they had the same protein concentration. The GlO1 activity was deduced using the following equations:

GlO1 activity (μmol min^−1^ mL^−1^) = (Abs 5 min − Abs 0 min)/5 min/2.86 mM^−1^ cm^−1^ 100 (dilution factor, DF) (5)

GlO1 specific activity = GlO1 activity/protein content = (μmol min^−1^ mL^−1^)/mg/mL. (6)

### 2.21. Amadori Product Assay

The HSFs were seeded into 6-well plates and treated as described in Section 2.2. The cells were collected and lysed with RIPA (containing 1% PMSF) and placed on ice. The experiment was performed following the instructions of the glycosylated serum protein assay kit (NBT–colorimetric method) (Beijing Solarbio Science & Technology Co., Ltd.).

### 2.22. Statistical Analyses

Data are presented as the means ± S.D. Statistical significance was evaluated by a Student’s *t*-test or one-way ANOVA where appropriate, and *p*-values < 0.05 were considered as significant. All experiments were performed at least three times.

## 3. Results

### 3.1. Establishment of an HG-Induced Glycation Model in HSF

#### 3.1.1. Effects of Different Concentrations of Glucose on HSF

**Figure 1:**
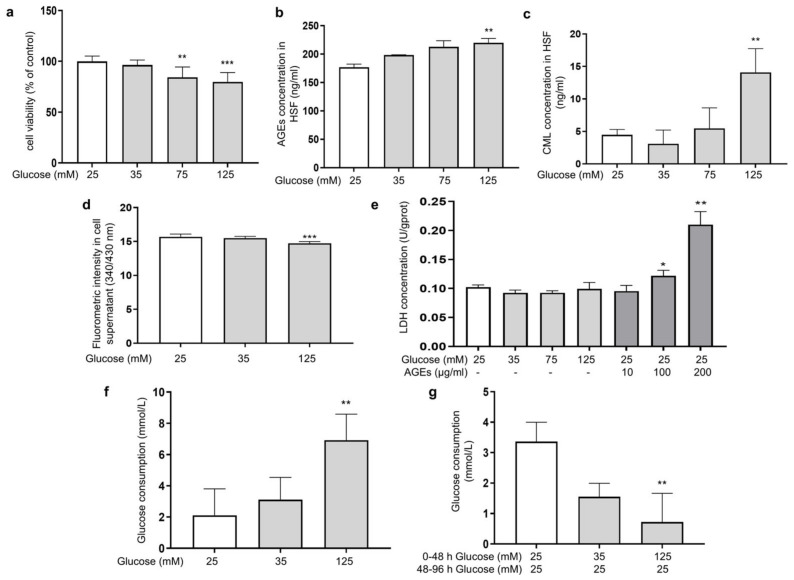
HG-induced intracellular glycation in HSFs. (**a**) The viability of HSFs cultured with (25, 75, and 125 mM) glucose for 48 h; (**b**) the intracellular AGEs concentration in HSFs cultured with (25, 75, and 125 mM) glucose for 48 h.; (**c**) the intracellular CML concentration in HSFs cultured with (25, 75, and 125 mM) glucose for 48 h; (**d**) fluorescent intensity of the cell supernatant cultured with (25, 75, and 125 mM) glucose for 48 h; (**e**) the LDH concentration in the culture medium after HSFs were cultured with 35, 75, and 125 mM glucose or AGEs (10, 100, 200 μg/mL) for 48 h; (**f**) glucose consumption of HSFs during 48 h of incubation in 25, 35, and 125 mM of glucose; (**g**) glucose consumption of HSFs during the second 48 h incubation, which were initially incubated in 25, 35 m and 125 mM of glucose for 48 h and then re-cultured in a fresh DMEM medium for another 48 h. * *p* < 0.05, ** *p* < 0.01, *** *p* < 0.001 vs. control group (25 mM of glucose). (n = 4).

Glucose plays a prominent role as an energy source for cell growth and state maintenance, but it is also the cause of adverse reactions, such as the production of glycation products. In order to investigate the effect of different glucose concentrations on the glycation reaction in fibroblasts, HSFs were cultured in DMEM supplemented with glucose at final concentrations of 25, 35, 75, and 125 mM for 48 h. Here, 25 mM of glucose was chosen as the normal condition. The MTT results showed that the concentration of glucose at 75 and 125 mM significantly inhibited cell viability in a dose-dependent manner (Figure 1a), indicating that HG could induce damage to HSFs. Since HSFs are the major collagen-secreting cells, both intracellular and extracellular glycation may happen under HG conditions. To clarify this, we analyzed the intracellular levels of AGEs and CML (biomarkers of the glycation process [38]), as well as the fluorescence intensity in the supernatant as an indication of the content of fluorescent AGEs in the culture fluid. The results showed that 125 mM of glucose significantly increased the AGEs content and intracellular CML by 24.2% and 213.5%, respectively, compared to the control group (Figure 1b,c), while the fluorescence intensity in the culture fluid of the HG group decreased compared to the low-dose glucose and the control group (Figure 1d), indicating that HG mainly caused intracellular glycation in the HG-induced HSF model.

Some previous studies pointed out that an increased release of lactate dehydrogenase (LDH) was found when cell membrane proteins were modified by AGEs [39]. Therefore, we experimented upon HSFs with different concentrations of AGEs. Compared to the control group, both 100 μg/mL and 200 μg/mL of AGEs significantly increased the amount of LDH outflow in the culture medium by 19% and 105.4%, respectively. However, this phenomenon was not observed in the HG groups (Figure 1e). This indicates that under the HG conditions, the AGEs generated in the medium were insufficient to affect the cell membrane structure, further confirming that the glycation events induced by HG occurred predominantly intracellularly.

To further examine this phenomenon, we measured the changes in glucose concentration in the culture medium. The results showed that the glucose consumption of the HG groups after being cultured for 48 h was higher than that in the low-dose group (Figure 1f), suggesting that HG promoted glucose uptake by HSFs to some extent, thereby inducing intracellular metabolism and glycation-related events. To examine whether the HG uptake was sustained, the glucose content in the medium was measured after the cells were incubated with HG (35 mM and 135 mM) for 48 h, then replaced with fresh DMEM medium (25 mM of glucose) and allowed to culture for another 48 h. The results showed that the glucose uptake was significantly suppressed in the HG-induced HSFs, with a 78.3% decrease in glucose consumption in the 125 mM of glucose-injured group compared to the control group (Figure 1g). This suggests that glucose uptake initially increased when fibroblasts were under HG conditions. However, the more glucose that was ingested, the more glycolysis and ROS were produced and the more reactions with intracellular proteins occurred. All of this leads to intracellular oxidative and glycolytic damage.

#### 3.1.2. HG Induces Inflammation and Oxidative Stress and Promotes Premature Cellular Senescence in HSFs

**Figure 2:**
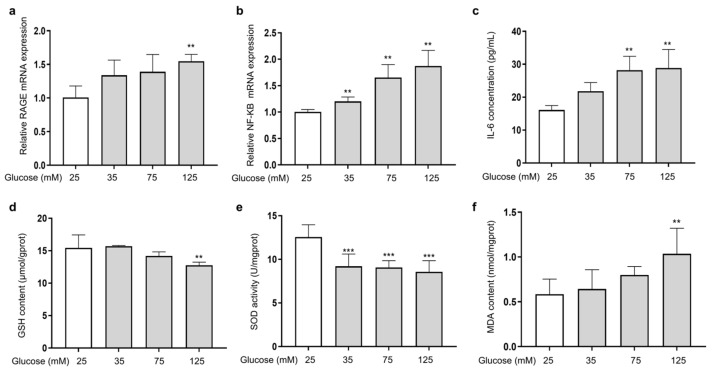
HG-induced cellular inflammation and oxidative stress in HSFs. HSFs were cultured with 25, 75, and 125 mM of glucose for 48 h. The basal medium contained 25 mM of glucose as the control group. (**a**) The relative RAGE mRNA expression level in HSFs; (**b**) the relative NF-κB mRNA expression level in HSFs. (**c**) The IL-6 concentration in the cell supernatant; (**d**) the GSH content in HSFs. (**e**) The SOD activity in HSFs; (**f**) the MDA content in HSFs. ** *p* < 0.01, *** *p* < 0.001 vs. control group (25 mM of glucose). (n = 4).

The ability of AGEs to bind to the RAGE receptor on the cell membrane and activate the transcription factor kappa B (NF-κB) is a major damage mechanism that promotes the release of inflammatory factors [27]. To investigate the effect of HG on the expression of *RAGE* and *NF-κB* in HSFs, the mRNA expression of *RAGE* and *NF-κB*, along with the secretion of the inflammatory factor IL-6 (one of the main pro-inflammatory factors [40]), were measured. The results showed that as the glucose concentration increased, the mRNA expression of *RAGE* (Figure 2a) and *NF-κB* (Figure 2b) increased compared to the control group. Meanwhile, IL-6 secretion was also increased (Figure 2c). HG and AGEs both accelerate intracellular ROS production and induce cellular oxidative stress [41]. Therefore, analyzing the levels of glutathione (GSH) and superoxide dismutase (SOD), which are related to antioxidant ability of cells, alongside the level of malondialdehyde (MDA), which reflects the extent of oxidation damage to the cells, is essential. The results showed that as the glucose concentration increased, the cellular GSH content (Figure 2d) and SOD (Figure 2e) activity were reduced, and the cellular MDA content (Figure 2f) increased compared to the control group. The above results show that HG could induce cell inflammation by activating RAGE receptors and downstream NF-κB signaling. In addition, excessive glucose levels could cause intracellular oxidative stress, diminish cellular antioxidant capacity, and intensify intracellular glycation.

**Figure 3:**
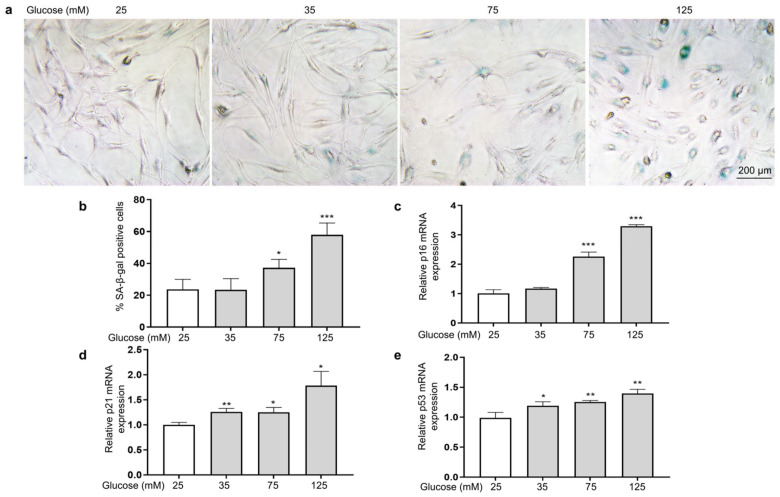
HG-induced HSF senescence. HSFs were cultured with 25, 75, and 125 mM of glucose for 48 h. The basal medium contained 25 mM of glucose as the control group. (**a**) Representative photographs of SA-β-gal staining (×200); (**b**) statistical results of SA-β-gal-positive cells; (**c**) the relative p21 mRNA expression level in HSFs; (**d**) the relative p16 mRNA expression level in HSFs. (**e**) The relative p53 mRNA expression level in HSFs. * *p* < 0.05, ** *p* < 0.01, *** *p* < 0.001 vs. control group (25 mM of glucose). (n = 4).

To investigate the effect of HG on the senescence of HSFs, we examined indicators of cellular senescence, including senescence-associated β-galactosidase (SA-β-gal), p16, p21, and p53 [42]. The results show that as the glucose concentration increased, the number of senescent cells (SA-β-gal positive cells appear blue) increased (Figure 3a,b), and the mRNA expression levels of p16 (Figure 3c), p21 (Figure 3d), and p53 (Figure 3e) were also significantly increased.

**Figure 4:**
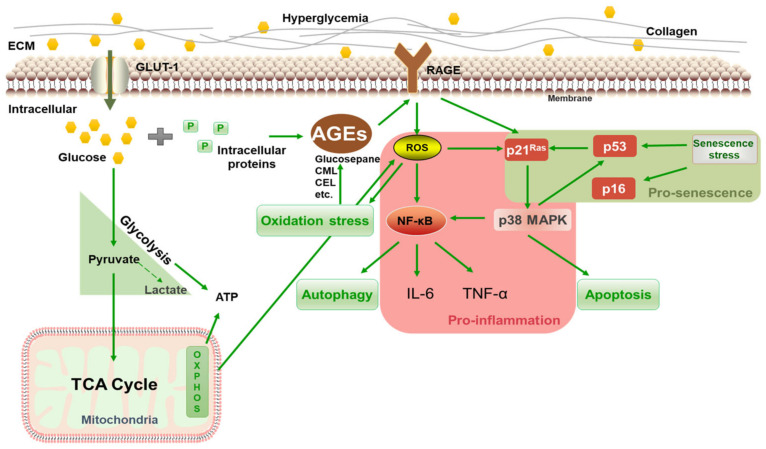
Schematic diagram of HG-induced intracellular glycation.

The above results showed that 125 mM of glucose effectively induced HSF intracellular glycation, resulting in a significant increase in the glycation marker CML, which upregulated RAGE and NF-κB mRNA expression, caused cellular inflammation and oxidative stress, and eventually promoted the premature senescence of cells. Thus, 125 mM of glucose was chosen as the model condition for inducing glycation in HSFs. The glycation process is illustrated in Figure 4. Under high glucose concentrations, glucose continuously enters cells through the GLUT1 pathway [43] and reacts with proteins inside the fibroblasts, generating AGEs via the Maillard reaction and activating the membrane receptor RAGE, triggering various signaling events, such as inflammation, oxidative stress, apoptosis, and autophagy. HG also increases oxidative stress and AGE generation by activating glucose self-oxidation and polyol metabolism.

### 3.2. Study on the Anti-Glycation, Anti-Inflammatory, Anti-Senescent, and Antioxidant Efficacy of LSE

#### 3.2.1. Preliminary Anti-Glycation and Antioxidant Effects of LSE

**Figure 5:**
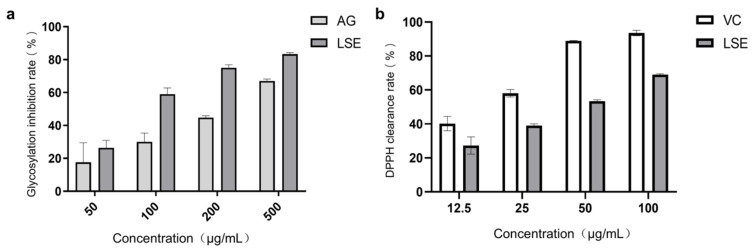
The anti-glycation and antioxidation ability of LSE in vitro. (**a**) The glycation inhibition effecta of AG and LSE in the BSA–fructose reaction system (n = 5); (**b**) The DPPH clearance rates of VC and LSE in vitro (n = 3).

Lotus stamens contain a variety of flavonoids, and have antioxidation, anti-aging, and other pharmacological activities [44,45]. The total flavonoids (TFC) and total polyphenols (TPC) were the main indexes to investigate the extraction conditions of lotus stamens. Here, 30% ethanol was chosen ultimately as the extraction solvent. The extraction rate of lotus stamens was 23.2%, and the TPC and TFC of LSE were 26.5% and 21.4%, respectively. For preliminary studies, a BSA–fructose in vitro reaction system and DPPH assay were conducted using aminoguanidine (AG) and VC as positive controls, respectively. The results showed that the non-enzymatic glycosylation inhibition effect of LSE was better than that of AG (Figure 5a). Both glucose oxidation and Amodori products’ self-oxidation led to the production of AGEs and free radical superoxide production, which means antioxidation also contributes to anti-glycation [46]. The DPPH clearance rate showed that the IC_50_ of VC was 17.38 μg/mL, while the IC_50_ of LSE was 41 μg/mL (Figure 5b), indicating that LSE exhibited good antioxidant efficacy.

#### 3.2.2. Anti-Glycation Effects of LSE on an HG-Induced HSF Glycation Model

**Figure 6:**
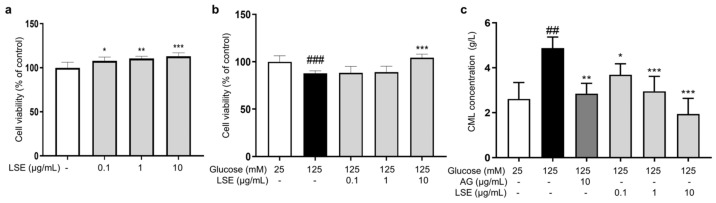
The protective and anti-glycation effects of LSE on the HG-induced HSFs. (**a**) The viability of HSFs cultured with 0.1, 1, and 10 μg/mL LSE for 48 h; (**b**) the viability of HSFs was assayed after incubation with LSE (0.1, 1, and 10 µg/mL) for 2 h followed by 125 mM of glucose for another 48 h; (**c**) the intracellular CML concentration in HSFs was assayed after incubation with AG (10 µg/mL) and LSE (0.1, 1, and 10 µg/mL) for 2 h, followed by 125 mM of glucose for another 48 h. ^##^ *p* < 0.01, ^###^ *p* < 0.001 vs. control group (25 mM of glucose); * *p* < 0.05, ** *p* < 0.01, *** *p* < 0.001 vs. model group (125 mM of glucose). (n = 4).

Subsequently, we examined the anti-glycation effect of LSE in the HG-induced HSF glycation model. The MTT results demonstrated that LSE was not cytotoxic to HSFs and could promote cell proliferation in the 0.1–10 μg/mL concentration range (Figure 6a). Additionally, LSE showed a protective effect towards the damage induced by HG. The 10 μg/mL LSE group increased cell viability by 18.6% compared to the control group (Figure 6b). AG is an AGE inhibitor that captures reactive carbonyl precursors (e.g., MGO, GO, and 3-DG) in vitro, but has toxicity at higher concentrations [47]. Our experiment results showed that both AG and LSE could significantly inhibit intracellular CML contents (Figure 6c), thereby demonstrating their anti-glycation effects in the HG-induced HSF glycation model.

#### 3.2.3. Effect of LSE on HG-Induced Senescence and Glycation-Related mRNA Expression in HSFs

**Figure 7:**
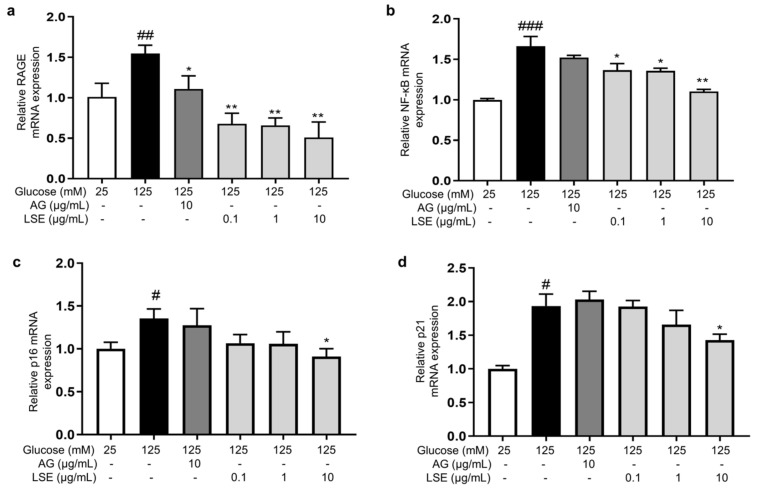
The effects of LSE on the expression of mRNAs related to HG-induced cellular senescence and glycation in HSFs. HSFs were treated with AG (10 µg/mL), LSE (0.1, 1, and 10 µg/mL), or AG (10 µg/mL) for 2 h, then incubated with 125 mM of glucose for 48 h. The relative mRNA expression levels of *RAGE* (**a**), *NF-κB* (**b**), p16 (**c**), and *p21* (**d**) were assayed. ^#^ *p* < 0.05, ^##^ *p* < 0.01, ^###^ *p* < 0.001 vs. control group (25 mM of glucose); * *p* < 0.05, ** *p* < 0.01 vs. model group (125 mM of glucose). (n = 4).

**Figure 8:**
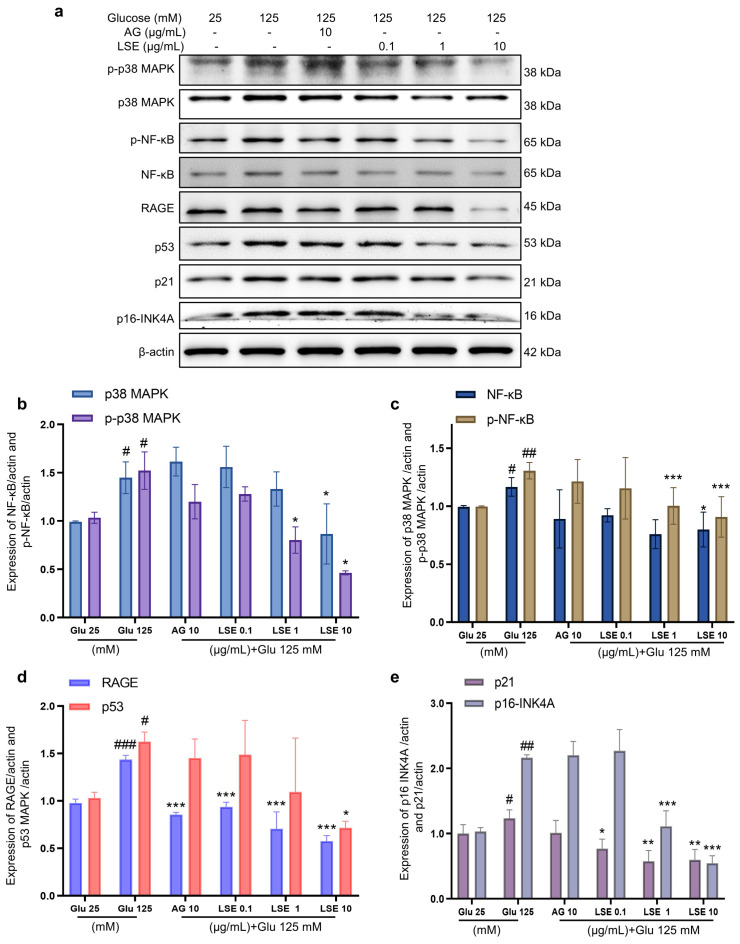
The effects of LSE on protein levels related to HG-induced cellular senescence and glycation in HSFs. HSFs were treated with AG (10 µg/mL), LSE (0.1, 1, and 10 µg/mL), or AG (10 µg/mL) for 2 h, then incubated with 125 mM of glucose for 48 h. The images of protein bands (**a**) and the relative protein levels of p38 MAPK (**b**), p-p38 MAPK (**b**), NF-κB (**c**), p-NF-κB (**c**), RAGE (**d**), p53 (**d**), p21 (**e**), and p16-INK4A (**e**) were analyzed. ^#^ *p* < 0.05, ^##^ *p* < 0.01, ^###^ *p* < 0.001 vs. control group (25 mM of glucose); * *p* < 0.05, ** *p* < 0.01, *** *p* < 0.001 vs. model group (125 mM of glucose). (n = 3).

To investigate the anti-inflammatory and anti-senescent effects of LSE on the HSF-induced HSF glycation model, the mRNA expression and protein levels of *RAGE*, *NF-κB*, *p16*, and *p21* mRNA were measured. The results showed that LSE could suppress the upregulation of *RAGE*, *NF-κB*, *p16*, and *p21* mRNA expression induced by HG in a concentration-dependent manner (Figure 7a–d), as well as the protein levels of p38 MAPK, p-p38-MAPK, NF-κB, p-NF-κB, RAGE, p53, p21, and p16-INK4A (Figure 8a–e). The above results indicate that LSE can inhibit HG-induced HSF inflammation and cellular senescence.

#### 3.2.4. Antioxidant Effects of LSE on the HG-Induced HSF Glycation Model

**Figure 9:**
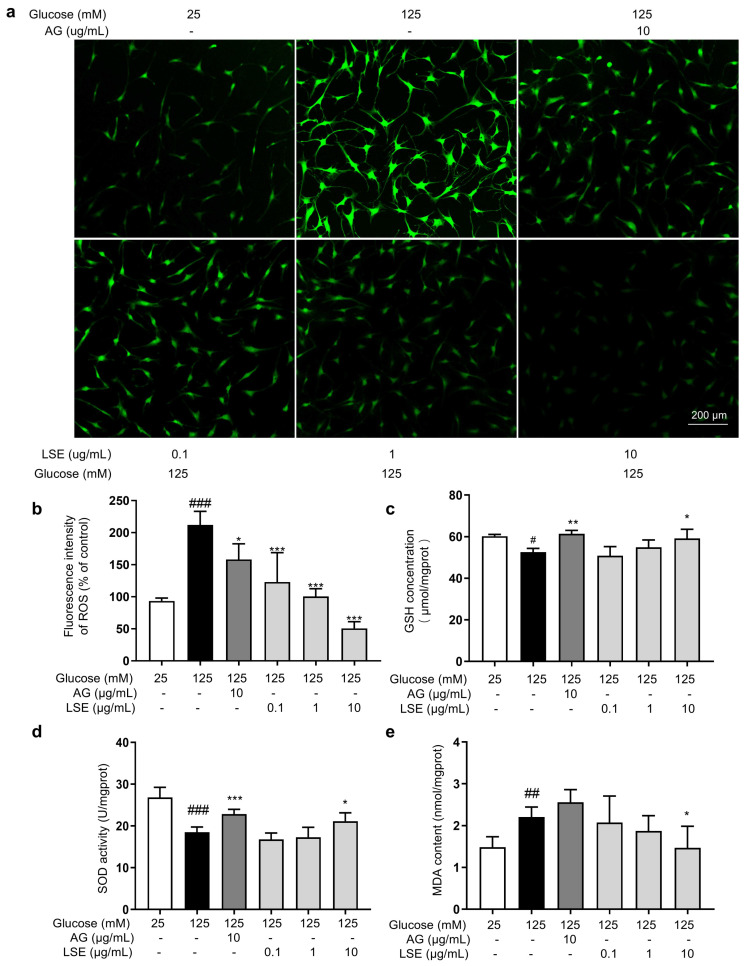
The antioxidant effects of LSE on the HG-induced HSFs. HSFs were pretreated with AG (10 µg/mL), LSE (0.1, 1, and 10 µg/mL), or AG (10 µg/mL) for 2 h, then incubated with 125 mM of glucose for 48 h. (**a**) Representative photographs of ROS fluorescent staining; (**b**) statistical results of ROS fluorescence intensity. (**c**) SOD activity in HSF; (**d**) GSH content in HSF; (**e**) MDA content in HSFs. ^#^ *p* < 0.05, ^##^ *p* < 0.01, ^###^ *p* < 0.001 vs. control group (25 mM of glucose); * *p* < 0.05, ** *p* < 0.01, *** *p* < 0.001 vs. model group (125 mM of glucose). (n = 4).

Oxidative stress also plays an important role in the formation of AGEs, and reducing oxidative stress helps reduce AGE generation. Therefore, the antioxidant effects of LSE in the HG-induced HSF glycation model were investigated. The results showed that in this model, LSE significantly reduced intracellular ROS fluorescence intensity in a concentration-dependent manner, increased cellular GSH content and SOD activity, and decreased MDA content compared to the model control (Figure 9). The above results show that LSE can alleviate the oxidative stress induced by HG and contribute to reducing the production of AGEs in cells to some extent.

### 3.3. Identification of Flavonoids in LSEE by HPLC-MS/MS

**Figure 10:**
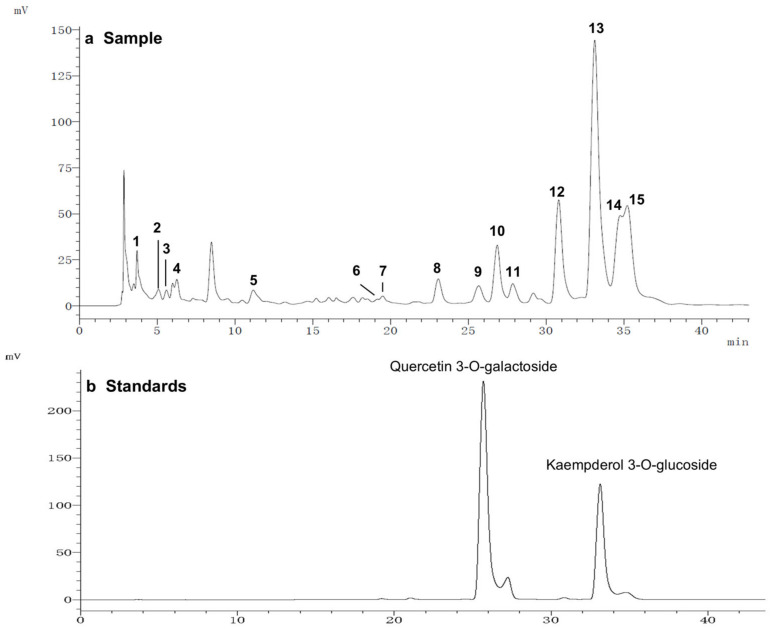
HPLC chromatograms of (**a**) LSEE and (**b**) a mixture of standard flavonoids. HPLC and MS/MS data of LSEE, including retention time, molecular ion in the negative ion mode (NI), aglycone ions, and important fragment ions, are listed in Table S1. The MS and MS/MS spectra for compounds **1**–**15** are shown in Figures S3–S7.

**Figure 11:**
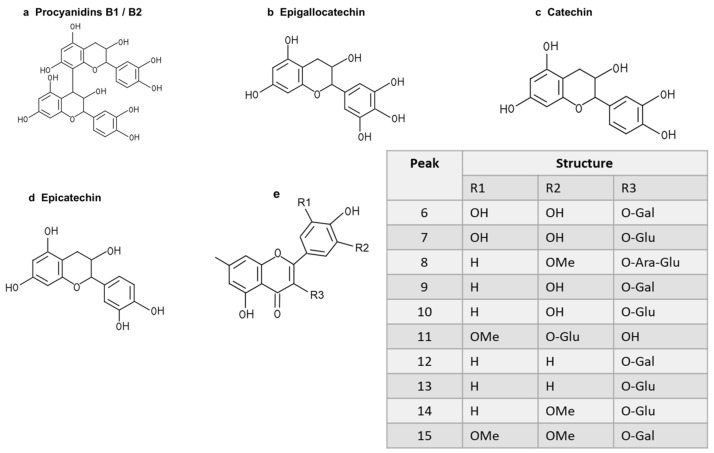
Structures of flavonoids and O-glycosides found in LSEE. (**a**) Structure of procyanidin B1 or B2; (**b**) structure of epigallocatechin; (**c**) structure of catechin; (**d**) structure of epicatechin; (**e**) structural model of flavonoid O-glycosides found in LSEE. The following sugar abbreviations are used: Glu, glucoside; Gal, galactoside; Ara, arabinoside.

To further study the relationship between the composition of LSE and its anti-glycation effect, LSE was extracted using petroleum ether, ethyl acetate, and n-butanol in increasing order of polarity. The yields of different polar parts of LSE, anti-glycation effects in the BSA–fructose assay, DPPH clearance rates, and TPC and TFC results are shown in Figure S1. Among the extractions, the lotus stamen ethyl acetate extract (LSEE) showed the most significant anti-glycation effect. The components of LSEE were further identified by HPLC-MS/MS, with 15 peaks clearly detected and recognized (Figure 10a). Peaks 9 and 13 were validated by standards (Figure 10b), while other compounds were identified by comparing the MS and MS/MS data with the published literature. The HPLC and MS/MS data, including retention time, molecular ion in the negative ion mode (NI), aglycone ions, and important fragment ions for LSEE, are listed in Table S1 (the MS and MS/MS spectra of compounds **1**–**15** are shown in Figures S3–S7). In addition, the normalization method of peak area was used to calculate the relative content of individual flavonoids, which was also listed in Table S1. Most flavonoids in LSEE were flavonoid 3-O-glycosides (Figure 11e). Five types of flavonoids were identified in LSEE, including quercetin, kaempferol, myricetin, syringetin, and isorhamnetin. The most abundant flavonoids were kaempferol derivatives (kaempferol 3-O-galactoside and kaempferol 3-O-glucoside (astragalin)), accounting for approximately 47% of the total) and quercetin derivatives (quercetin 3-O-galactoside (hyperoside) and quercetin 3-O-glucoside (isoquercitrin)), accounting for approximately 9% of the total. LSEE also contained several oligomeric proanthocyanidins (OPCs), including proanthocyanin B1, proanthocyanin B2, epigallocatechin, catechin, and epicatechin, which are polyphenolic flavonoids (Figure 11a–d).

### 3.4. The Study of the Anti-Glycation Effect and Mechanism of LSEE and Its Key Flavonoids

**Figure 12:**
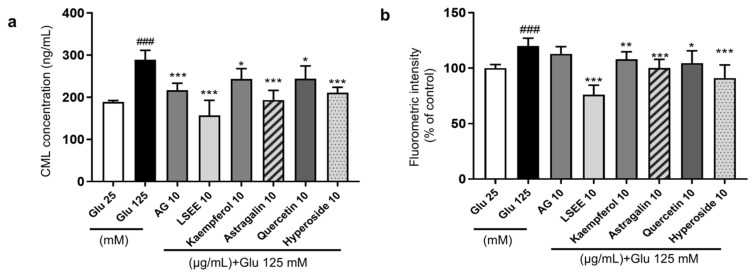
The anti-glycation effect of LSEE and flavonoids in HG-induced HSFs. HSFs were pretreated with 10 µg/mL of AG, LSEE, kaempferol, astragalin, quercetin, and hyperoside for 2 h, then incubated with 125 mM of glucose for 48 h. (**a**) The intracellular CML concentration in HSFs was measured by ELISA. (**b**) The fluorescence intensity of HSFs was measured by flow cytometry. ^###^ *p* < 0.001 vs. control group (25 mM of glucose); * *p* < 0.05, ** *p* < 0.01, *** *p* < 0.001 vs. model group (125 mM of glucose). (n = 4).

Flavonoids are a class of polyphenols categorized into flavonols, flavones, flavanones, isoflavones, catechins, anthocyanidins, and chalcones, and have significant inhibitory effects on AGE formation [48]. Quercetin is a common flavonol known for its strong antioxidant activity and is more effective than AG in inhibiting glycation at all stages [49]. Kaempferol is a polyphenol flavonoid that also possesses good antioxidant activity and can inhibit the generation of AGEs and AGE-related NF-κB signal cascades [50]. Studies have demonstrated that an increase in the number of hydroxyl groups and 3-glycosylation tends to enhance the AGE-inhibitory activity of flavonols [51]. In this study, two representative flavonoid glycosides, namely quercetin 3-O-galactoside (hyperoside) and kaempferol 3-O-glucoside (astragalin), were selected to investigate the anti-glycation effects in the HG-induced HSF glycation model. The results showed that both hyperoside and astragalin exhibited stronger anti-glycation effects than quercetin and kaempferol (Figure 12a). The cellular fluorescence intensity results, which represented the level of fluorescent AGEs, were obtained using flow cytometry. The fluorescence results were generally consistent with CML levels (Figure 12b). This suggests that the 3-O glycosylation modification of flavonoids may enhance their anti-glycation effects. Considering that kaempferol derivatives account for approximately 47% of LSEE, they are assumed to the main components driving the anti-glycation activity in LSEE.

### 3.5. LSEE and Its Key Flavonoids Promote GLO1 Expression Through the Nrf2/Keap1 Pathway

**Figure 13:**
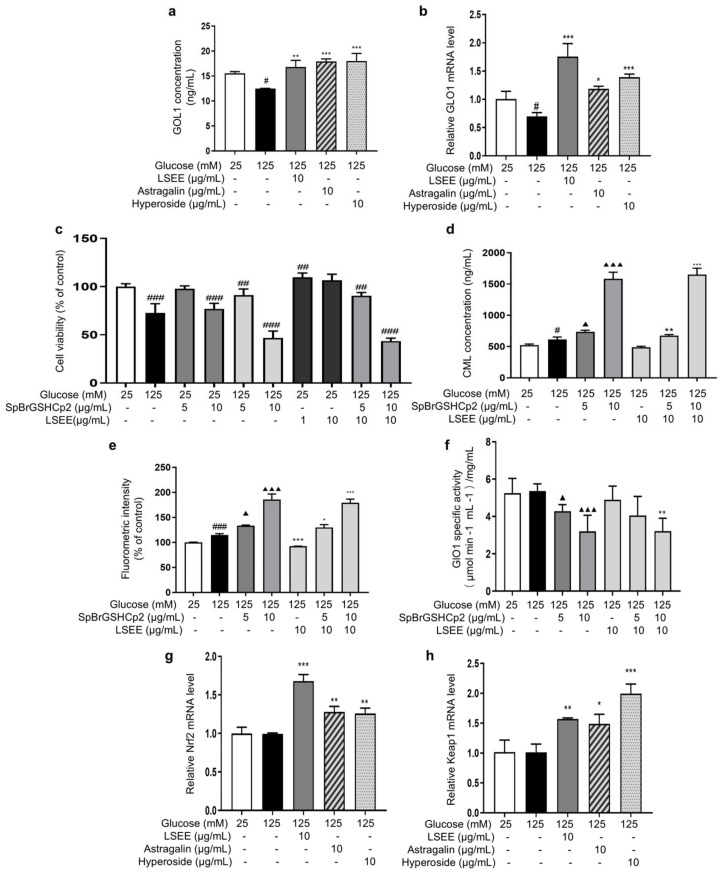
LSEE and its key flavonoids promote GLO1 expression through the Nrf2/Keap1 pathway. HSFs were pretreated with 10 µg/mL of LSE, astragalin, and hyperoside for 2 h, then incubated with 125 mM of glucose for 48 h. (**a**) The GLO1 concentration in HSFs was measured by ELISA. (**b**) The relative *GLO1* mRNA expression level. (**c**) The viability in HSFs injured by glucose (125 mM) for 48 h or injured by SpBrGSHCp2 (5 and 10 μg/mL) for 48 h or injured by both glucose (125 mM) and SpBrGSHCp2 (5 and 10 μg/mL) for 48 h, or pretreated with LSEE (1 and 10 µg/mL) for 48 h or pretreated with 10 µg/mL of LSEE for 2 h, then injured by both glucose (125 mM) and SpBrGSHCp2 (5 and 10 μg/mL) for 48 h; (**d**) The CML concentration in HSFs injured by glucose (125 mM) for 48 h or injured by both glucose (125 mM) and SpBrGSHCp2 (5 and 10 μg/mL) for 48 h, or pretreated with 10 µg/mL of LSEE for 2 h, then injured by both glucose (125 mM) and SpBrGSHCp2 (5 and 10 μg/mL) for 48 h. (**e**) The fluorescence intensity in HSFs injured by glucose (125 mM) for 48 h or injured by both glucose (125 mM) and SpBrGSHCp2 (5 and 10 μg/mL) for 48 h, or pretreated with 10 µg/mL of LSE for 2 h, then injured by both glucose (125 mM) and SpBrGSHCp2 (5 and 10 μg/mL) for 48 h. (**f**) The GLO1 enzymatic activity in HSFs injured by glucose (125 mM) for 48 h, or injured by both glucose (125 mM) and SpBrGSHCp2 (5 and 10 μg/mL) for 48 h, or pretreated with 10 µg/mL of LSEE for 2 h, then injured by both glucose 125 mM for 48 h, or pretreated with 10 µg/mL of LSEE for 2 h, then injured by both glucose (125 mM) and SpBrGSHCp2 (5 and 10 μg/mL) for 48 h. (**g**) The relative Nrf2 mRNA expression level in HSFs pretreated with 10 µg/mL of LSEE, astragalin and hyperoside for 2 h, then injured by glucose (125 mM) for 48 h. (**h**) The relative Keap1 mRNA expression level in HSFs pretreated with 10 µg/mL of LSEE, astragalin, and hyperoside for 2 h, then injured by glucose (125 mM) for 48 h. ^#^ *p* < 0.05, ^##^ *p* < 0.01, ^###^ *p* < 0.001 vs. control group (25 mM of glucose); * *p* < 0.05, ** *p* < 0.01, *** *p* < 0.001 vs. model group (125 mM of glucose); ^▲^ *p* < 0.05, ^▲▲▲^ *p* < 0.001 vs. model group (125 mM of glucose). (n = 3).

Normal physiological activity of cells and the degradation of Amadori products during the glycation process can produce a variety of highly reactive dicarbonyl compounds (MGO and GO), which are capable of causing 65% of glycation events [15]. The glyoxalase system consists of glyoxalase I and II (GlO1 and GlO2), which are the main enzymes involved in the detoxification of dicarbonyl stress. Reduced activity or expression of GlO1 will cause MGO or GO to accumulate in cells [52], which glycates intracellular proteins and mitochondria and affects cell structure and function [53]. In order to investigate the effects of LSEE, astragalin, and hyperoside on GLO1, the intracellular content and mRNA expression of GLO1 were detected. The results showed that HG decreased the intracellular GLO1 content and mRNA expression, while LSEE, astragalin, and hyperoside all significantly increased both the intracellular GLO1 content and mRNA expression compared to the HG group (Figure 13a,b).

Furthermore, a GLO1 inhibitor, namely S-p-Bromobenzylglutathione cyclopentyldiester (SpBrGSHCp2), was employed to investigate the anti-glycation mechanisms of LSEE, astragalin, and hyperoside. The addition of SpBrGSHCp2 to cultured cells causes intracellular MGO accumulation, leading to an increase in nucleotide glycation and inducing apoptosis [54]. MTT results showed that 10 μg/mL of SpBrGSHCp2 decreased cell viability by 23.1% compared to the control group. When 125 mM of glucose and SpBrGSHCp2 worked together, cell viability decreased by 53.2% compared to the control group, and the occurrence of apoptosis significantly increased (Figure 13c). This is consistent with the prediction that the addition of SpBrGSHCp2 would exacerbate the HG-induced increase in MGO accumulation, leading to massive apoptosis in HSFs. Additionally, pretreatment with LSEE did not improve the decrease in cell viability caused by combined injury from HG and SpBrGSHCp2. 

Subsequently, the effect of SpBrGSHCp2 on cellular glycation was investigated by measuring intracellular CML levels and cellular fluorescence intensity. The results showed that 125 mM of glucose significantly increased intracellular CML by 17.9 %, and that the CML concentration increased by 204.3% when 125 mM of glucose and 10 μg/mL SpBrGSHCp2 worked together. Meanwhile, 10 μg/mL LSEE decreased CML by 20.6% compared to the HG model group, but the anti-glycation effect of LSEE disappeared following the addition of SpBrGSHCp2 (Figure 13d). The fluorescence results were basically consistent with CML levels (Figure 13e). Further investigation demonstrated that intracellular GLO1 activity decreased by 18.4% and 39% at 5 and 10 μg/mL of SpBrGSHCp2, respectively, compared to the control group. LSEE treatment did not enhance the activity of GlO1 inhibited by SpBrGSHCp2 (Figure 13f). These results suggest that GLO1 plays an important role in cellular anti-glycation, and promoting GLO1 expression is the main anti-glycation mechanism of LSEE, which would block by SpBrGSHCp2. We also detected glucose consumption, mitochondrial membrane potential, cellular autophagy, Amodori products, and interactions between LSEE and proteins. The results showed that LSEE could interact with intracellular proteins and decrease the quantity of Amodori products (Figure S2).

The Nrf2/Keap1/ARE pathway is an important signaling pathway involved in the equilibrium regulation of redox and is an important part of the body’s antioxidant defense system [55]. GlO1 is a downstream target of the Nrf2/Keap1 pathway when it exerts its protective function, and the activation of Nrf2 may enhance GLO1 function, thereby alleviating dicarbonyl stress [56]. In order to investigate the effect of LSEE on the Nrf2/Keap1 pathway, the mRNA expression of Nrf2 and Keap1 were measured. The results showed that 125 mM of glucose had no effect on Keap1 and Nrf2 mRNA expression. However, LSEE, astragalin, and hyperoside all significantly increased the mRNA expression of Keap1 and Nrf2 (Figure 13g,h). Therefore, LSEE and its key flavonoids may increase GLO1 expression through the activation of the Keap1/Nrf2 path-way, thereby improving the antioxidant and anti-glycation effects in HG-induced HSFs.

## 4. Discussion

The most common method used in anti-glycation research is the BSA–fructose reaction system, which can easily obtain the non-enzymatic glycosylation inhibition rate of testing samples and compare the anti-glycation ability of them [57]. If these components have effects of binding free amino acids or reduced sugars, removing free radicals or capturing dicarbonyl compounds, they will show a certain anti-glycation effect as long as the combination of free amino acid groups is prevented. Therefore, some common dietary products, such as yoghurt, millet [58], tea [59], and fruit [60,61] show an anti-glycation effect. However, it is unknown whether these products can also work in the human body as anti-glycation agents. Some diabetes studies use mice as test subject to detect Amadori glycated protein in serum (e.g., HbA1c is the Amadori product of hemoglobin and glucose) [62], fluorescence in the skin, CML, and RAGE expression levels in different tissues to characterize the degree of glycation [63]. Diabetic skin shows elevated matrix metalloproteinases (MMPs) and lysyl oxidase (LOX) levels on dermis that lead to fragmentation and the accumulation of cross-linked collagen, thus resulting in the aging phenotype in the skin of diabetics [64]. As a common glycation damage agent, HG can influence fibroblast collagen level by decreasing fibroblast *TIMP-2* mRNA expression and increasing the expression of multiple MMPs, including *MMP-1* and *MMP-2*. In addition, HG can also decrease fibroblast vitality, inducing cell inflammation [65] and apoptosis and promoting the premature senescence of cells [66]. However, more research is needed on AGE formation and glycation-related events in fibroblasts exposed to HG. Therefore, a simpler and more reliable model to reflect glycation-related events in cells is crucial for anti-glycation studies.

In this study, we investigated the impact of different concentrations of glucose (25 mM, 35 mM, 75 mM, and 125 mM) on fibroblasts, and a glucose concentration of 125 mM was selected as the treatment condition for the glycation model. At this concentration, fibroblasts are sensitive to reproducing the generation of intercellular glycation markers [67], CML, and glycation-associated events, such as RAGE, ROS, inflammation, and cellular senescence markers. This is particularly significant for studying diabetes and its complications, such as microvascular disease [68], nerve damage, and related conditions [69,70,71]. Based on this model, we observed that fibroblasts initially increased glucose uptake in a HG environment, but subsequently exhibited damage and senescence phenotypes, accompanied by a decrease in glucose uptake. It can be assumed that elevated glucose level leads to high glycolysis and tricarboxylic acid cycle activity, which is associated with both more ROS and glycation-related events/injury. However, a glucose concentration of 125 mM is much higher than the physiological glucose levels in the blood of normal individuals (5.6–6.9 mM). Some studies have shown that the relatively high glucose concentration commonly used in cell culture media to support rapid cell growth may affect the experimental results [72,73]. Therefore, future research should consider designing experiments based on the physiological glucose levels of diabetic patients and extending the stimulation time to investigate the impact on fibroblast glycation.

Anti-aging is a complex concept involving various physiological processes, particularly the mechanisms of cellular senescence. Cellular senescence is typically understood as a self-protective response that cells undergo in response to prolonged exposure to external stimuli and detrimental conditions, in order to prevent potential malignant transformation or irreversible damage [74]. A HG environment has been proven to be one of the key factors inducing cellular senescence, especially in fibroblasts [75,76,77]. The oxidative stress, glycation reactions, and inflammation caused by HG together accelerate the senescence process. Therefore, we investigate the anti-senescent effects of LSE in this process. Our study demonstrated that LSE significantly downregulates the upregulation of *p16* and *p21* mRNA expression induced by HG, both of which are crucial proteins in cell cycle regulation and play a key role in cellular senescence [78]. Western blot experiments further confirmed that as the concentration of LSE increased, LSE could inhibit the excessive expression of p16, p21, and p53 proteins, as induced by HG. These results suggest that LSE has the potential to delay the cellular senescence process and that it improves the function and state of the cells. The anti-senescence effects of LSE may stem from the synergistic regulation of oxidative stress, inflammation, and glycation reactions by its flavonoid components. By scavenging free radicals, slowing down glycation reactions, and inhibiting inflammation, the active components in LSE can effectively reduce the damage caused by HG and delay the onset of senescence. This finding consistent with current research trends on the anti-senescence mechanisms of plant extracts, which emphasize the crucial role of plant active ingredients in multi-target anti-senescence. For example, Garcinia flavonoids have antioxidant and anti-inflammatory properties and activate cellular defense mechanisms, making them a natural anti-senescence agent [79]. Bamboo leaf flavonoids (BLF) delay skin aging by exerting antioxidant and anti-inflammatory effects, activating the p38 MAPK signaling pathway and promoting autophagy [80].

Our study found that in the HG-induced HSF glycation model, different polarity fractions of LSE, including the water phase (LSEW), butanol phase (LSEB), and ethyl acetate phase (LSEE), all exhibited anti-glycation activities. However, the overall effect of LSE was significantly superior to that of any individual fraction. Further comparisons revealed that the anti-glycation activity of LSE was also notably higher than that of two flavonoid monomers, namely hyperoside (quercetin-3-O-galactoside) and astragalin (kaempferol-3-O-glucoside). This phenomenon suggests that the synergistic interactions of multiple components in LSE may be more effective than a single component, which is consistent with findings that the synergistic potential of various herbs and therapeutic agents in health improvements [81]. For instance, a study on corn zein protein aqueous extract demonstrated a synergistic enhancement of GLP-1 secretion in intestinal endocrine cells, mediated by γ-aminobutyric acid (GABA) and L-phenylalanine (Phe) [82]. In addition, the fermentation extract of Aloe arborescens breaks down larger-molecular-weight polysaccharides into smaller molecules. When combined with its antioxidant component, barbaloin, a synergistic effect is generated, which significantly enhances its anti-wrinkle activity on the skin [83]. Similarly, the coextracted proteins (COP) from Ginseng and Schisandra exhibit synergistic effects, significantly enhancing their antioxidant activity and cellular protective functions [84].

However, the complexity of the chemical component in plant extracts and the heterogeneity of raw material sources may lead to fluctuations in experimental results. Variations in batch, origin, or preparation methods can influence the content and ratio of active components, thereby causing instability in anti-glycation efficacy. Therefore, elucidating the composition–activity relationship is essential to ensure the reproducibility of the experimental results. Our study demonstrated that the flavonoid constituents in LSEE may significantly enhance the expression of GLO1 by activating the Nrf2/Keap1 pathway, an antioxidant defense system [85,86], thus accelerating the metabolism and clearance of dicarbonyl compounds and playing a key regulatory role in inhibiting cellular glycation. Therefore, ensuring the stability of key flavonoid markers, such as quercetin-3-O-galactoside and kaempferol-3-O-glucoside, in the extract would allow for reliable verification of the anti-glycation activity of LSE. This finding supports the development of a standardization system for plant extracts based on the quantitative control of active components.

## 5. Conclusions

Our study demonstrated that HG induces intracellular glycation reactions, as evidenced by increased levels of CML and cellular fluorescence intensity, accompanied by oxidative stress, inflammation, and premature senescence. LSE exhibits anti-glycation effects in the HG-induced HSF glycation model and also shows antioxidant, anti-inflammatory, and anti-senescence potential. The primary anti-glycation mechanism is the activation of the Nrf2/Keap1 pathway, which leads to an increased expression of GLO1. GLO1 is involved in alleviating the dicarbonyl stress induced by HG, thereby inhibiting cellular glycation. This was experimentally confirmed by both LSEE and its representative compounds, hyperoside (quercetin-3-O-galactoside) and astragalin (kaempferol-3-O-glucoside). These findings strongly support the anti-glycation ability of flavonoid constituents in LSE and clarify the direct connection between anti-glycation and antioxidant effects. Together, these results provide a solid foundation for the development of lotus stamens as a natural and organic ingredient for skin beauty and healthcare applications.

## Figures and Tables

**Table 1:** Gene primer sequence information.

Gene	Primer Sequence (5′-3′)		GenBank Accession Numbers
*β-actin*	Forward primer	5′-CTACCTCATGAAGATCCTCACCGA-3′	NM_001101
	Reverse primer	3′-TTCTCCTTAATGTCACGCACGATT-5′	
*p16*	Forward primer	5′-GAAGCCGGGGTTTCGCCCAA-3′	NM_000077
	Reverse primer	3′-GCACCGGGCGGGAGAAGGTA-5′	
*p21*	Forward primer	5′-ACATTCAGAGCCACAGGCACCA-3′	NM_000389
	Reverse primer	3′-GCATCGCAATCACGGCGCAA-5′	
*p53*	Forward primer	5′-CAGCCAAGTCTGTGACTTGCACGTAC-3′	NM_000546
	Reverse primer	3′-CGAAAAGTGTTTCTGTCATC-5′	
*RAGE*	Forward primer	5′-CACCTTCTCCTGTAGCTTCA-3′	NM_001301717
	Reverse primer	3′-TGCCACAAGATGACCCCAA-5′	
*NF-κB*	Forward primer	5′-TCATGAAGAAGAGTCCTTTCAGC-3′	NM_001042292
	Reverse primer	3′-GGATGACGTAAAGGGATAGGG-5′	
*GLO1*	Forward primer	5′-ATGCGACCCAGAGTTACCAC-3′	NM_002062
	Reverse primer	3′-CCAGGCCTTTCATTTTACCA-5′	
*Nrf2*	Forward primer	5′-ACACGGTCCACAGCTCATC-3′	NM_006164
	Reverse primer	3′-TGTCAATCAAATCCATGTCCTG-5′	
*Keap1*	Forward primer	5′-GACTGGGTCAAATACGACTGC-3′	NM_003594
	Reverse primer	3′-GAATATCTGCACCAGGTAGTCC-5′	

## Data Availability

Data is contained within the article and Supplementary Materials.

## Supplementary Materials

The following supporting information can be downloaded at: https://www.mdpi.com/article/10.3390/antiox14040392/s1, Table S1: Identification and the relative content of 15 flavonoids in lotus stamen extraction ethyl acetate by HPLC-MS/MS. The peak numbers correspond to those used in Figure 10a; Figure S1: The anti-glycation effect of different polar fractions of LSE; Figure S2: The anti-glycation effect and mechanism of LSEE on HG-induced HSFs; Figure S3: The MS and MS/MS spectra of (a,b) compounds 1/2 and (c,d) 3; Figure S4: The MS and MS/MS spectra of compounds (a,b) 4/5 and (c,d) 6/7; Figure S5: The MS and MS/MS spectra of compounds (a,b) 8 and (c,d) 9/10; Figure S6: The MS and MS/MS spectra of compounds (a,b) 11 and (c,d) 12/13; Figure S7: The MS and MS/MS spectra of compounds (a,b) 14 and (c,d) 15.

## Abbreviations

The following abbreviations are used in this manuscript:

AGEs	Advanced glycation end products
HG	High-glucose
HSFs	Human skin fibroblasts
LSE	Lotus stamen extract
LSWE	Lotus stamen water extract
LSBE	Lotus stamen n-butanol extract
LSEE	Lotus stamen ethyl acetate extract
LSPE	Lotus stamens petroleum ether extract
RAGE	The receptor of advanced glycation end products
CML	Nε-(carboxymethyl) lysine
CEL	Nε-(carboxyethyl) lysine
GO	Glyoxal
MGO	Methylglyoxal
3-DG	3-deoxyglucosone
MG-H1	MGO-derived hydroimidazolone
MOLD	Methylglyoxal-lysine dimer
G-H1	GO-derived hydroimidazolone
GOLD	Glyoxal-lysine dimer
MAPK	Mitogen-activated protein kinase
IL-6	Interleukin 6
TNF-α	Tumor-necrosis factor alpha
MMPs	Matrix metalloproteinases
MMP1	Matrix metalloproteinase 1
MMP2	Matrix metalloproteinase 2
MMP9	Matrix metalloproteinase 9
ROS	Reactive oxygen species
BSA	Bovine serum albumin
HPLC	High-performance liquid chromatography
HPLC-MS/MS	High-performance liquid chromatography-tandem mass spectrometry
NI	Negative ion mode
DMSO	Dimethyl sulfoxide
ECM	Extracellular matrix
PVDF	Polyvinylidene fluoride
AG	Aminoguanidine
GLO1	Glyoxalase 1
LOX	Lysyl oxidase
LDH	Lactate dehydrogenase
MDA	Malondialdehyde
SOD	Superoxide dismutase
VC	L-ascorbic acid
OPCs	Oligomeric proanthocyanidins
Nrf2	Nuclear factor-erythroid 2-related factor 2
Keap1	Kelch-like ECH-associated protein 1
SpBrGSHCp2	S-p-bromobenzylglutathione cyclopentyldiester
BLF	Bamboo leaf flavonoids
GABA	γ-aminobutyric acid
Phe	L-phenylalanine
