# Supplementary material for: Flavonoids in Lotus Stamen Extract Inhibit High Glucose-Induced Intracellular Glycation in Fibroblasts by Upregulating the Expression of Glyoxalase 1 and Alleviating Oxidative Stress
Source: Antioxidants (Basel). 2025 Mar 26;14(4):392. doi: 10.3390/antiox14040392 (PMC12024171; doi:10.3390/antiox14040392)
Supplement: Supplementary file 1 [file antioxidants-14-00392-s001.zip › antioxidants-3493025-supplementary-update.pdf]

Supplementary materials

1. Identification and quantification of 15 flavonoids in LSEE by HPLC-MS/MS

Table S1. Identification and the relative content of 15 flavonoids in LSEE by HPLC-MS/MS. The peak numbers correspond to those used in Figure 10a.

Peak No	Retention time (min)	NI-MS	MS-MS	Identification	Relative content (%)	Molecular formula
1	3.71	577	407;289	Procyanidin B1	4.15	$C_{30}H_{26}O_{12}$
2	5.09	577	407;289	Procyanidin B2	1.54	$C_{30}H_{26}O_{12}$
3	5.60	305	287;261	(-)-Epigallocatechin	1.05	$C_{15}H_{14}O_{7}$
4	6.13	289	125；137；165；179	[+]-Catechin	2.24	$C_{15}H_{14}O_{6}$
5	11.18	289	125；137；165；179	[+]-Epicatechin	1.96	$C_{15}H_{14}O_{6}$
6	19.16	479	316	Myr-3-O-Gal	0.45	$C_{21}H_{20}O_{13}$
7	19.50	479	316	Myr-3-O-Glu	1.36	$C_{21}H_{20}O_{13}$
8	23.07	609	314；299	Iso-3-Ara-Glu	2.97	$C_{27}H_{30}O_{16}$
9	25.67	463	300	Que-3-O-Gal/Hyperoside	2.69	$C_{21}H_{20}O_{12}$
10	26.86	463	300	Que-3-O-Glu/Isoquercetrin	6.34	$C_{21}H_{20}O_{12}$
11	27.86	493	330；315	Iso-3'-O-Glu/ Myricomplanoside	2.48	$C_{22}H_{22}O_{13}$
12	30.81	447	284	Kae-3-O-Gal	12.11	$C_{21}H_{20}O_{11}$
13	33.13	447	284	Kae-3-O-Glu/Astragalin	34.73	$C_{21}H_{20}O_{11}$
14	34.77	477	314	Iso-3-O-Glu	9.44	$C_{22}H_{22}O_{12}$
15	35.22	507	344	Syr-3-O-Gal	16.51	$C_{23}H_{24}O_{13}$

Names of the major flavonoid O-glycosides in LSEE. Flavonoids abbreviations used: Myr, myricetin; Syr, syringetin; Que, quercetin; Kae, kaempferol; Iso, isorhamnetin. Sugar abbreviations used: Glu, glucoside; Gal, galactoside; Ara, arabinoside.

2. The anti-glycation effect of different polar parts of LSE in vitro

To further investigate the components of LSE with anti-glycation efficacy, LSE was extracted using petroleum ether, ethyl acetate and n-butyl alcohol respectively and screened by anti-glycation ability in vitro. The yields of different polar parts of the extraction were: water layer (LSWE) 53.3%, n-butanol layer (LSBE) 32%, ethyl acetate layer (LSEE) 13.7%, petroleum ether layer (LSPE) 1% approximately. In the BSA-fructose reaction system, the non-enzymatic glycosylation inhibition rates of 100 μg/mL LSWE, LSBE, LSEE and LSPE were 58.3%, 78.6%, 80.2% and 52.7% respectively. The results showed that the anti-glycation effect of LSEE was significantly better than that of the other parts (Figure S1a). The anti-glycation effect of the different polar parts was also verified on HG-induced HSF glycation model. The results showed that compared to the model group, the CML levels in fibroblasts treated with 10 μg/mL LSWE, LSBE, LSEE, and LSPE were decreased by 41.4%, 36%, 47.5%, and 17.2% respectively. LSEE also exhibited the best anti-glycation effect in the cellular glycation model (Figure S1b).

As antioxidation is a part of anti-glycation, the DPPH free radical clearance of the different polar parts was detected. The results showed that LSBE had the highest free radical clearance rate (the clearance rate of 100 μg/mL LSBE was 70.3%), followed by LSWE, LSEE and LSPE (Figure S1c). LSBE also exhibited the highest total flavonoid content (TFC) and total phenolic content (TPC), followed by LSEE, LSWE, and LSPE (Figure S1d). The above results indicate that LSEE has the best anti-glycation effect in the BSA-fructose reaction system and the HG-induced fibroblast glycation model. However, the DPPH clearance rate, TFC, and TPC of LSEE were not as high as those of LSBE, suggesting that LSEE has ingredient-based advantages. Therefore, further component analysis is needed to identify the key components in LSEE responsible for its anti-glycation effects.

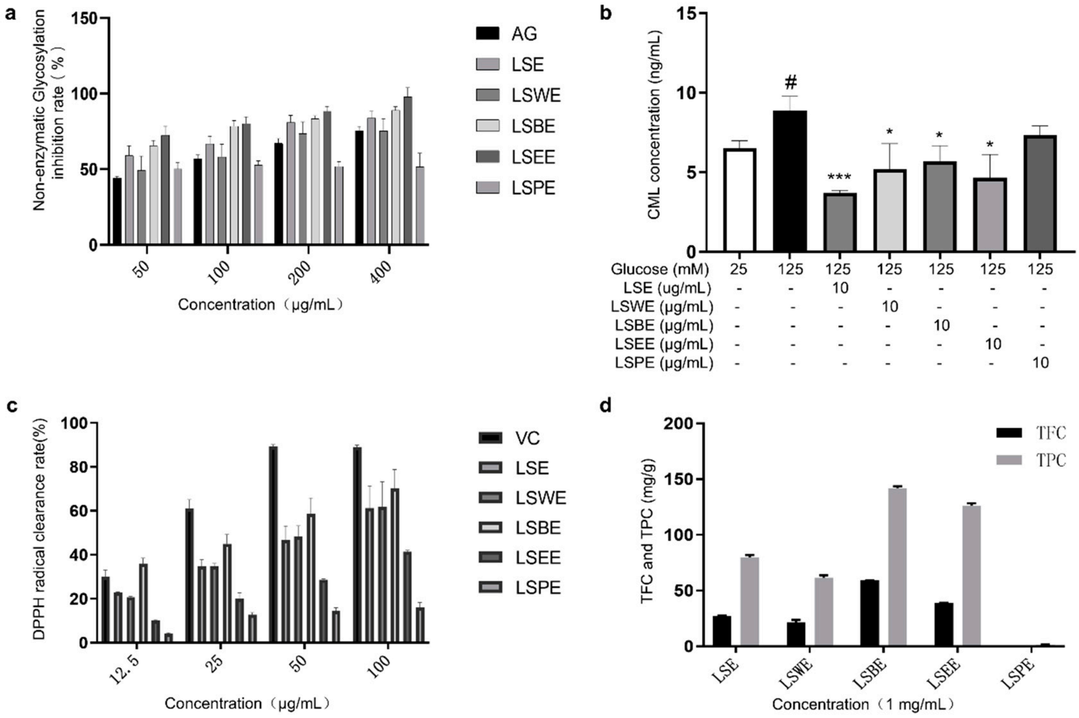

Figure S1. The anti-glycation effects of different polar fractions of LSE. (a) The non-enzymatic glycosylation inhibition rate of different polar fractions of LSE in the BSA-fructose system; (b) The CML concentration in fibroblast pretreated with 10 μg/mL of LSE, LSWE, LSBE, LSEE and LSPE for 2h, then incubated with glucose (125 mM) for 48h; (c) The DPPH clearance rate of different polar parts of LSE; (d) The TFC and TPC of different polar fractions of LSE in vitro. $^{\#}P < 0.05$ vs control group; $^{*}P < 0.05$, $^{***}P < 0.001$vs model group. ($n = 4$).

3. Mechanism study of LSEE on HG-induced HSF glycation

Studies have shown that flavonoids typically have multiple phenolic hydroxyl groups in their structures, which interact with serum proteins through hydrogen bonding, hydrophobicity, and π-π accumulation to form stable complexes that influence the glycation process. To investigate the role of LSEE in interacting with BSA on the formation of AGEs, a BSA-fructose reaction system was used to compare the non-enzyme glycosylation inhibition rate of LSEE-BSA pre-mixing, LSEE-fructose pre-mixing and fructose-BSA pre-mixing. The results showed that the LSEE-BSA pre-mixing exhibited a

higher non-enzyme glycosylation inhibition rate (Figure S2a). Additionally, the presence of serum in the LSEE administration reduced its anti-glycation effect, and this reduction was inversely proportional to the serum concentration (Figure S2b). Indicating that serum may interact with the flavonoids in LSEE, thereby reducing the ability of LSEE to enter cells and exert its effects. The Amadori products are the result of the first phase of protein non-enzyme glycosylation, formed by the rearrangement of an imine intermediate (Schiff base) between the amino group on the protein or the N-terminal amino group and the reduced sugar. The results showed that both AG and LSEE significantly reduced the intracellular Amadori products compared to the model group (Figure S2c). Therefore, LSEE may reduce the production of Amadori products by protecting intracellular proteins, thereby exhibiting certain anti-glycation activity.

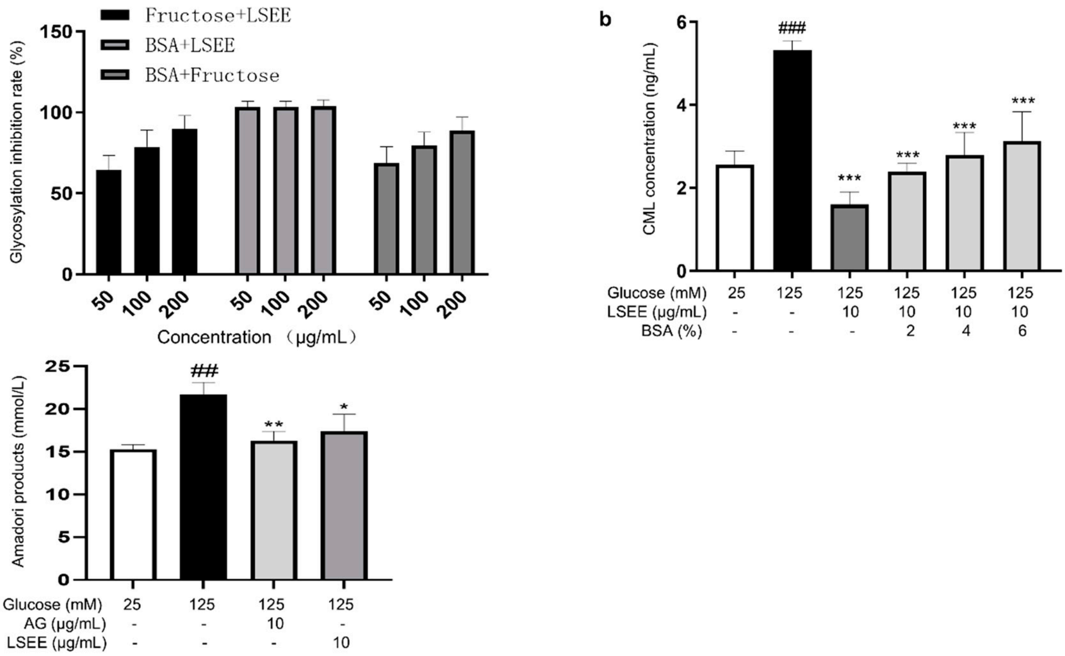

Figure S2. The anti-glycation effects and mechanism of LSEE on HSF-induced HSF glycation. (a) The effect of LSEE on the glycosylation inhibition rate in vitro BSA-fructose system; (b) The intracellular CML concentration in HSF was assayed after incubation with 10 μg/mL LSE for 2 h, followed by 125 mM glucose (with different concentrations of serum for another 48h; (c) The Amadori products in HSF was measured after pretreated with 10 μg/mL of AG and LSE for 2 h, then incubated with 125 mM glucose for 48 h. $^{\#\#}P < 0.01$, $^{\#\#\#}P < 0.001$ vs control group; $^{*}P < 0.05$, $^{***}P < 0.01$, $^{***}P < 0.001$vs model group. ($n = 4$).

4. MS and MS/MS Spectra of Compounds 1–15

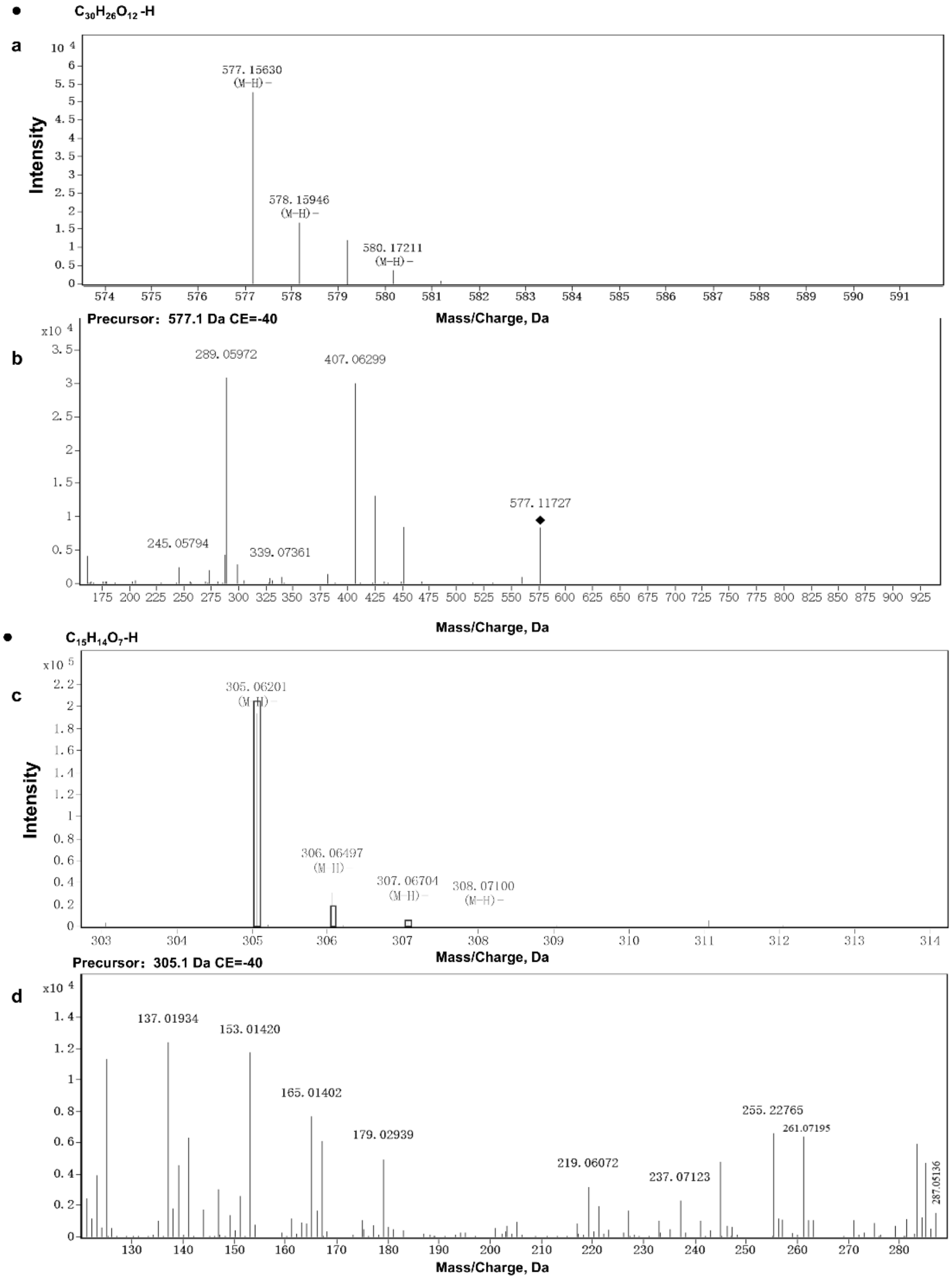

Figure S3. The MS and MS/MS spectrum of (a, b) compound 1/2 and (c, d) 3.

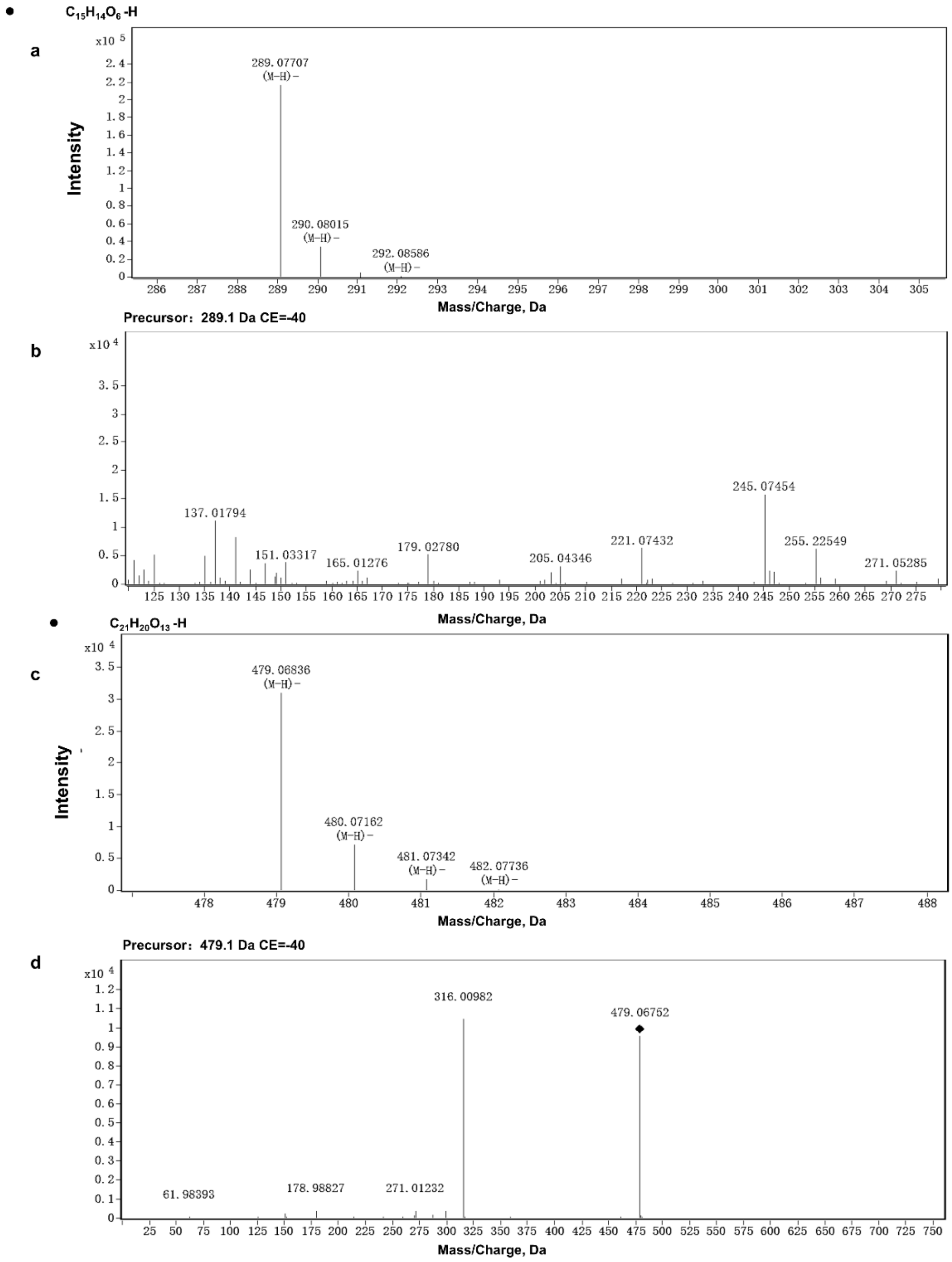

Figure S4. The MS and MS/MS spectrum of compound (a, b) 4/5 and (c, d) 6/7.

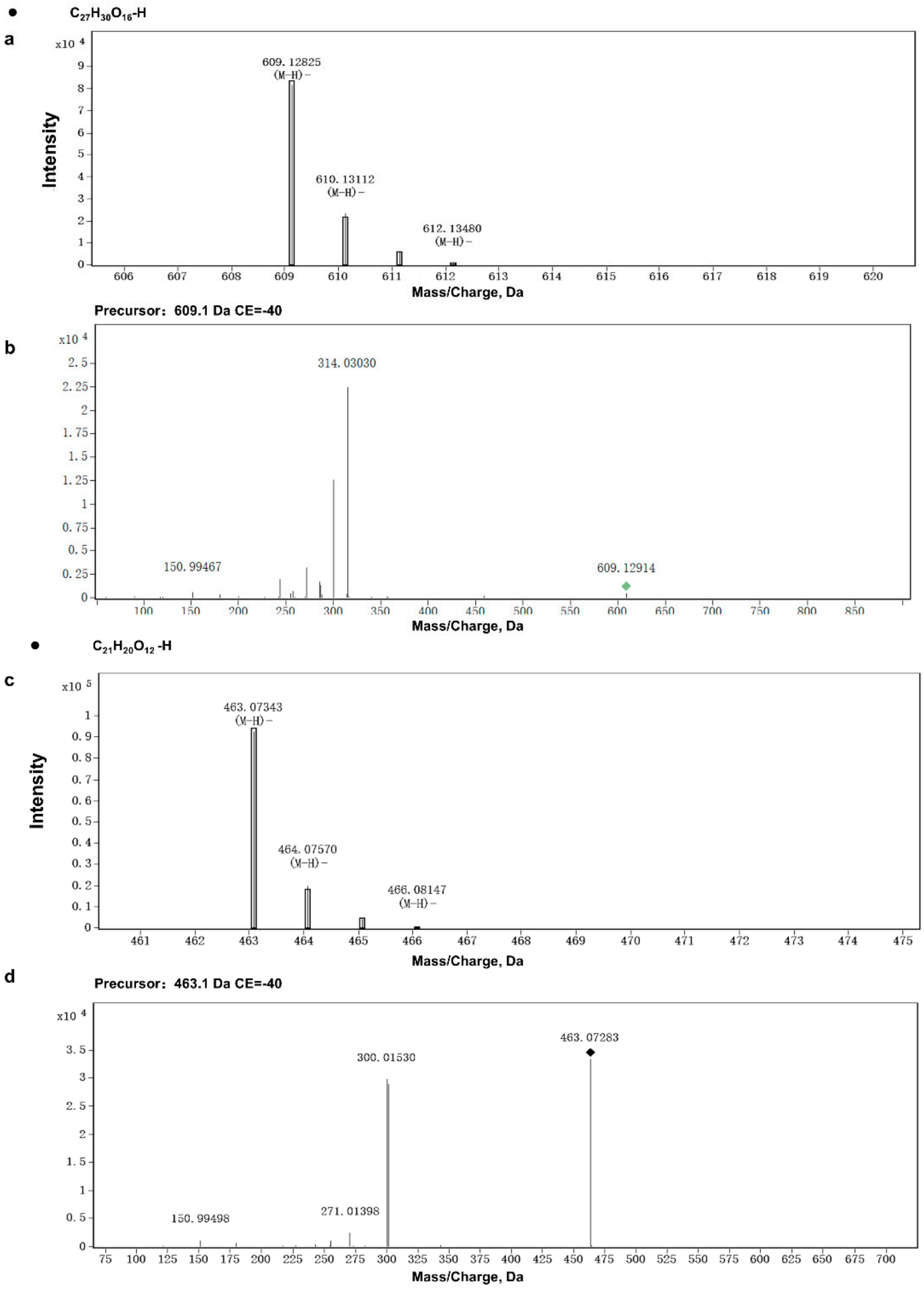

Figure S5. The MS and MS/MS spectrum of compound (a, b) 8 and (c, d) 9/10.

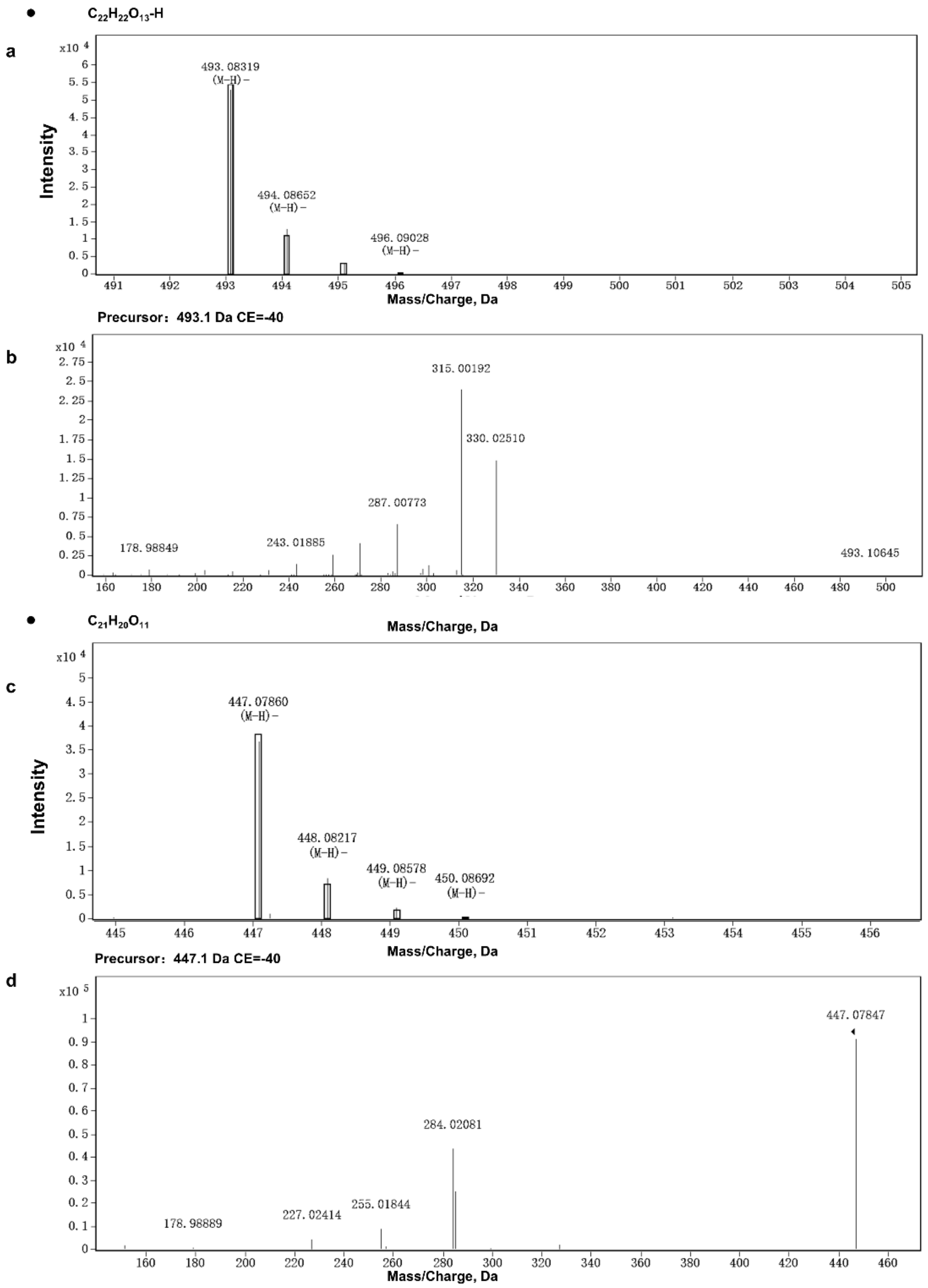

Figure S6. The MS and MS/MS spectrum of compound (a, b) 11 and (c, d) 12/13.

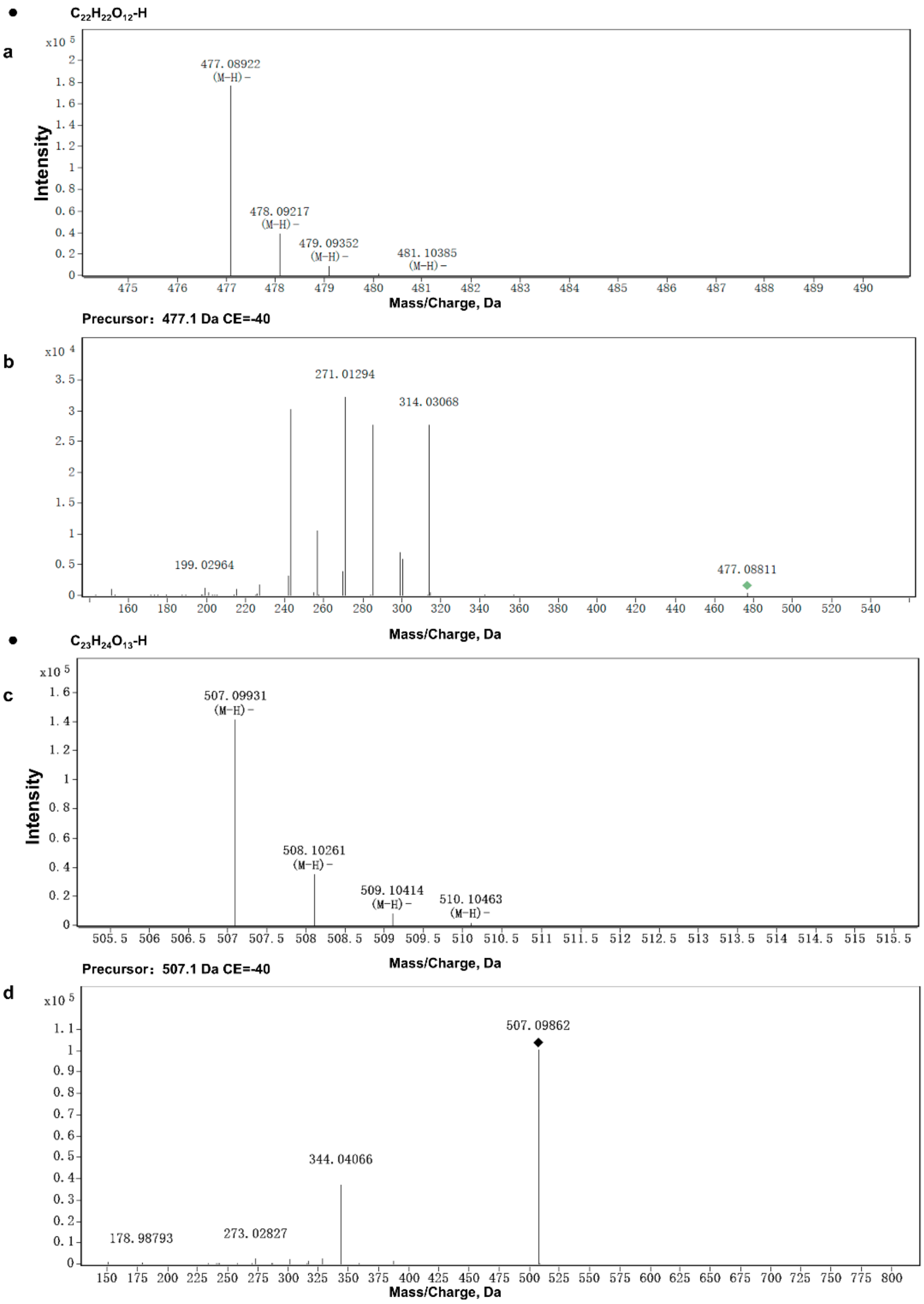

Figure S7. The MS and MS/MS spectrum of compound (a, b) 14 and (c, d) 15.
